# Synaptic ring attractor: A unified framework for attractor dynamics and multiple cues integration

**DOI:** 10.1016/j.heliyon.2024.e35458

**Published:** 2024-08-05

**Authors:** Yani Chen, Lin Zhang, Hao Chen, Xuelong Sun, Jigen Peng

**Affiliations:** a Machine Life and Intelligence Research Center, Guangzhou University, Guangzhou, China; b School of Mathematics and Information Science, Guangzhou University, Guangzhou, China

## Abstract

Effective cue integration is essential for an animal's survival. The ring attractor network has emerged as a powerful framework for understanding how animals seamlessly integrate various cues. This network not only elucidates neural dynamics within the brain, especially in spatial encoding systems like the heading direction (HD) system, but also sheds light on cue integration within decision-making processes. Yet, many significant phenomena across different fields lack clear explanations. For instance, in physiology, the integration mechanism of Drosophila's compass neuron when confronted with conflicting self-motion cues and external sensory cues with varying gain control settings is not well elucidated. Similarly, in ethology, the decision-making system shows Bayesian integration (BI) under minimal cue conflicts, but shifts to a winner-take-all (WTA) mode as conflicts surpass a certain threshold. To address these gaps, we introduce a ring attractor network with asymmetrical neural connections and synaptic dynamics in this paper. A thorough series of simulations has been conducted to assess its ability to track external cues and integrate conflicting cues. The results from these simulations demonstrate that the proposed model replicates observed neural dynamics and offers a framework for modeling biologically plausible cue integration behaviors. Furthermore, our findings yield several testable predictions that could inform future neuroethological research, providing insights into the role of ring attractor dynamics in the animal brain.

## Graphical abstract

**Figure:**
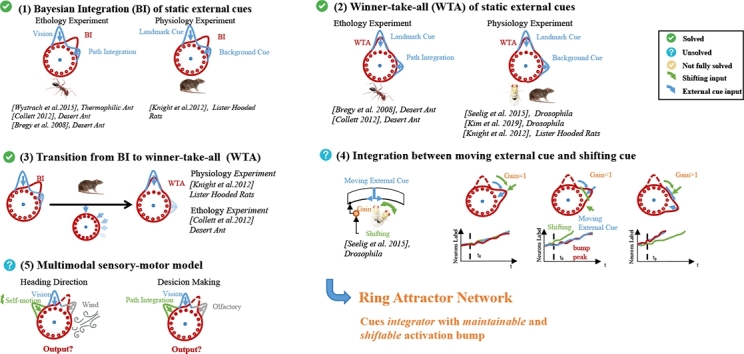

## 1 Introduction

**Figure 1:**
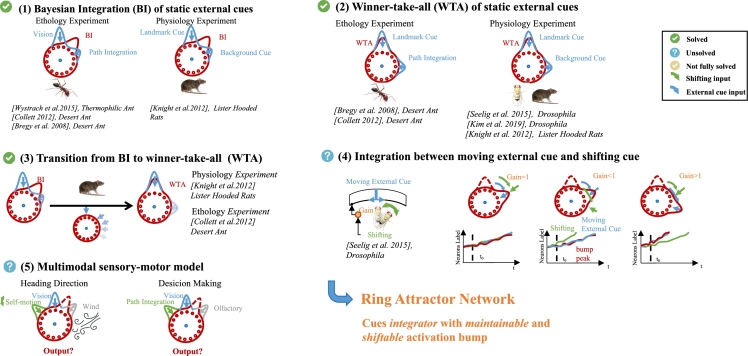
**Summary of biological experiment** The model inputs can be categorized into two distinct classes (Fig. 2): endogenous inputs (‘shifting’, e.g. self-motion, represented in green) and exogenous inputs (‘external cue’, e.g. vision, olfactory et al., represented in blue). The latter category includes both moving and static cues. This paper explores the cue integration, cue competition, and cue tracking, supported by significant biological experimental results presented in the figure. More broadly, ring attractor networks exhibit versatility beyond their conventional roles in HD systems or decision-making system, encapsulating the concept of a multi-modal cue integrator capable of maintaining and shifting activation peaks.

A fundamental challenge for animals is how to integrate information from multiple sensory modalities to achieve an accurate perception of their surroundings, ultimately enhancing their chances of survival. In physiology, research has demonstrated that the heading direction (HD) system ([1], [2], [3], [4], [5], [6]) can integrate cues from various sensory domains, such as vision [7], [8], [9], olfactory and mechanosensory [10], [11], [12] to dynamically represent the internal heading direction (Fig. 1). Ethological investigations also reveal the integration of multiple cues in the navigational decision-making processes of insects [3], [13], [14], [15], [16], [4], [5].

The basic mechanisms and computations underlying efficient cue integration, akin to those observed in animals, have captivated researchers for many years. Among the various approaches proposed to address this phenomenon, the *ring attractor network* has garnered significant attention. This network is characterized by translational invariance in neuronal interactions and has been widely applied to model the head direction (HD) systems of insects [17], [18], [19], [20] and rodents' [21], [22], [23], [24], [25], [26], [27], [28] HD systems. Moreover, the ring attractor network also offers a robust framework for weighted cue averaging integration within decision-making processes [29], [30], [31].

Despite advancements, some significant neural dynamics and properties remain unexplained (as summarized in Table 1 and Fig. 1). For instance, (1) the response of *Drosophila*'s compass neuron when it receives self-motion cue and external sensory cue (e.g., the moving visual cues displayed on the LED screen) with different degrees of coupling (i.e., the gain control [8]); (2) the head direction (HD) system exhibits Bayesian integration when conflicts among cues are minor, but transitions to a winner-take-all mode as conflict increases to a certain value [3]. Similarly, such switching behaviors have been reported in ethology simulations of insects [13], [14]. The underlying mechanism of this switching behavior remains unclear, and there is a lack of quantitative analysis regarding the threshold of conflicts that trigger such switches. Current computational models employing ring attractor networks have primarily focused on the simultaneous processing of only two cues [29], [32], [33], leaving questions about their ability to integrate a broader range of cues open.

**Table 1:** **Summary and comparison of previous work.** The following symbols are used to indicate the model's performance on specific questions: ✓ denote questions successfully resolved by the model, ✗ denote questions beyond the model's resolution capacity, and “Not Fully” denotes partially resolved questions. (1) Ring attractor model; (2) Investigating the critical conditions for the transition in a ring attractor network from cue integration to WTA; (3)-(5) Assessing the performance of activation integration (I) and WTA in compass neurons when confronted with two or multiple external cues (C); (6) Evaluating the ring attractor network's capability in tracking shifting input (*ω*); (7) Analyzing the integration of shifting (*ω*) and external cue inputs (*I*^*ext*^).

Ref.	(1) RA Model	(2) Switch	(3) 2C-I	(4) 2C-WTA	(5) More Cues	(6) *ω*	(7) *ω* + *I*^*ext*^
[21]	Yes	✗	✗	✗	✗	✗	✗
[23]	Yes	✗	✗	✗	✗	✓	✗
[24]	Yes	✗	✗	✗	✗	✗	✗
[25]	NO	✗	✗	✗	✗	✓	✗
[18]	Yes	✗	✗	✗	✗	✓	✗
[19]	Yes	✗	Not Fully	✓	✗	✓	✗
[28]	Yes	✗	✓	✗	✗	✓	✗
[29]	Yes	✓	✓	✗	✗	✗	✗
[31]	Yes	Not Fully	✓	✗	✗	✗	✗
[48]	NO	✗	✓	✓	✗	✗	✗
[51]	Yes	✗	Not Fully	✗	✗	✗	✗
[52]	NO	✗	✓	✗	Not Fully	✗	✗

**Figure 2:**
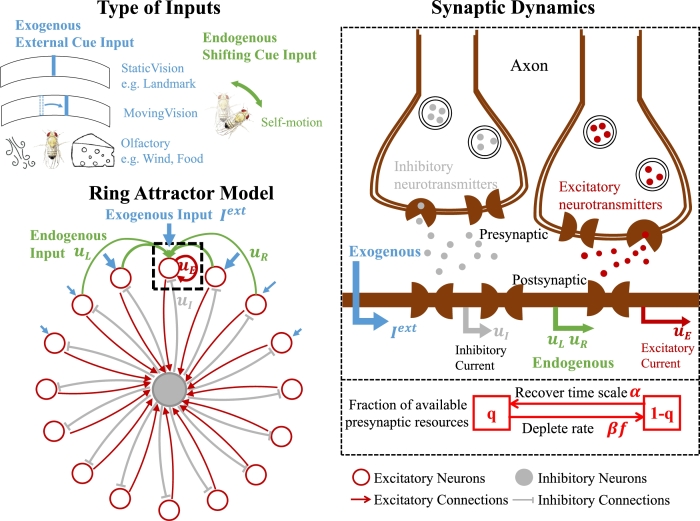
**The proposed ring attractor network with synaptic processing.** There are two types of inputs (exogenous and endogenous) in our model to regulate the system. Within the ring attractor model, excitatory neurons are recurrently connected in a ring-like arrangement (functional rather than necessarily physical), and they connected to a single inhibitory neuron. The synaptic mechanisms are governed by the availability of presynaptic resources denoted as *q*, which deplete at a rate given by *βf* and recover over a time scale specified by *α*.

To bridge these gaps, we developed a ring attractor model (see Fig. 2) incorporating asymmetrical neural connections [23] and multi-time scale neural synaptic dynamics [34], [35]. Mathematically, this model enriches the system's dynamical behaviors, including equilibria, relaxation cycles, and traveling waves, among other behaviors. These diverse dynamic behaviors enable the description of various phenomena such as tracking and selection. From a neuroscientific standpoint, incorporating synaptic processing into the neuron model holds great promise for enhancing the accuracy and realism of neurobehavioral descriptions.

To validate the capabilities of our proposed ring attractor model for cue integration, we conducted systematic simulations involving various types and quantities of input cues. The findings illustrate the model's adeptness in capturing key features of ring attractors, especially within HD systems, as observed in animal studies (as summarized in Fig. 1). Furthermore, the model effectively integrates diverse cues and provides an analysis of cue conflict thresholds that trigger transitions from integration to winner-take-all (WTA) dynamics.

Our investigation sheds light on the neural dynamics of ring attractors, emphasizing their potential as cue integrators and their relevance to intricate neural network functions like memory and learning.

## 2 Results

To systematically investigate the impact of incorporating synaptic processing into a ring attractor network, our model maintains identical neural structures and connections to those employed in numerous prior studies [21], [22], [23], [24], [25], [17], [18], [19], [26], [27], [36], [28]. In a typical ring attractor network, excitatory neurons are recurrently connected in a ring-like arrangement (functional rather than necessarily physical), and are connected to a uniform inhibitory neuron (see the left panel of Fig. 2). But in the proposed model, available presynaptic resources are regulated through synaptic processing (see the right panel of Fig. 2), with the parameter *α* representing the recovery speed [34], [35], [37]. The formulation of the proposed model is detailed in Methods section.

The subsequent subsections present the simulation results of the proposed model, which aims to reproduce the recognized dynamics of ring attractors observed in biological organisms. It is important to note that during these simulations:

1. Synaptic current *u* serves as indicator of neuronal activity [38], [26], [39], [27], [40]. While both positive and negative currents are included in the calculations, the figures presented throughout the paper emphasize only the positive current segments.
2. Various non-linear functions, such as the Heaviside step function, could alternatively be used as activation functions within the network. As both the sigmoid and Heaviside step functions exhibit similar asymptotic behavior, the choice of activation function may not substantially affect the stability properties of the system as discussed in this paper.
3. The activation bump (or hill) refers to the bell-shaped curve that characterizes the activation of excitatory neurons within the ring attractor.
4. Our model incorporates two types of inputs: 1) *External cue input (exogenous)* ($I^{ext}$ in Equation (1)), e.g. vision [7], [8], [9], olfactory and mechanosensory cues [10], [11], [12]. This input is directly integrated into the model. In our simulations, the external cue input can be dynamic, such as the moving visual stimuli displayed on the LED arena described by [8], or static, as seen in cue integration experiments reported by [15], [3]; 2) *Shifting input (endogenous, proprioceptive feedback)* (*ω* in Equation (5)), e.g. self-motion [8], which modulates the direction-determined neural connection strength.
5. The external cue input is modeled as a Gaussian function [1], [41], [23] N(μ,σ), where *μ* represents the peak position (the preferred direction of the corresponding neuron) and *σ* refers to the certainty of the cue [15], [29]. A static cue is represented by a constant *μ*, whereas a time-varying *μ* indicates a moving Gaussian-like cue. It is important to note that the terms *moving* and *traveling* are distinctly used to describe input and output, respectively. In the continuous limit, the stationary state of the system manifests as a continuous manifold. Within this manifold, the system exhibits neutral stability, enabling the network state to transition smoothly in response to changes in the shape of the external stimulus. (e.g. square-wave, Fig. S7, Fig. S8).
6. The presented results depict the neuron outputs only when the system reaches equilibrium. The appearance of equilibrium indicates the stability of the system in the absence of additional inputs. We selected a snapshot arbitrarily to characterize the neurons' response after reaching this equilibrium state.

### 2.1 Ring attractor with maintainable and shift-able activity bump

In the heading direction system of insects and rodents, the neural representation of compass orientation can be maintained even in the absence of inputs [8], [42], [43], [44], and it can be adjusted according to different initial states as well [8], [1]. Our proposed model effectively demonstrates and validates these two critical functions of neural networks responsible for directional representation.

#### 2.1.1 Maintaining the activity bump without external inputs

**Figure 3:**
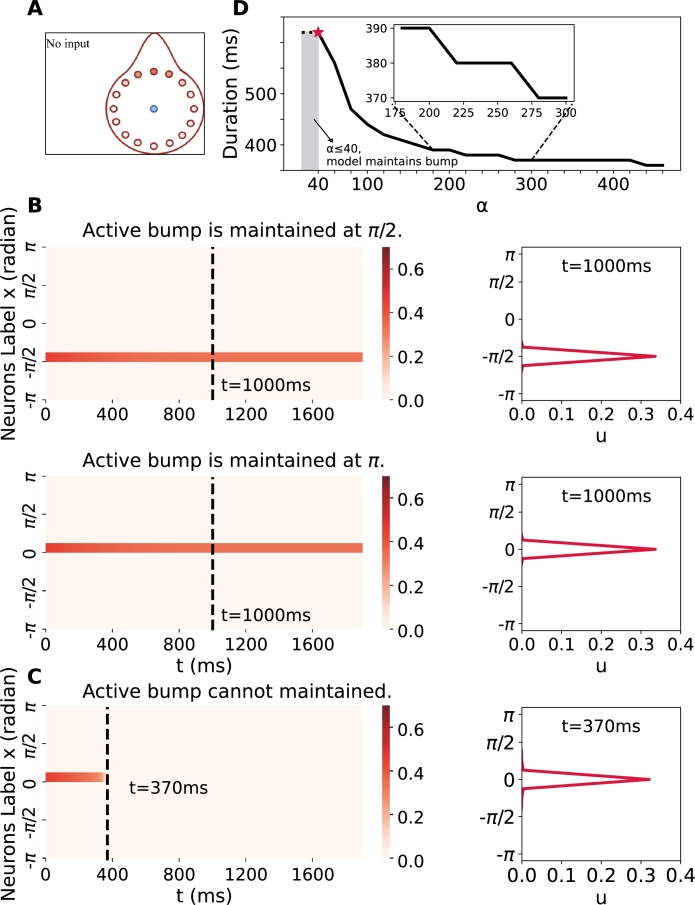
**Self-sustained neural persistent activity without inputs.** A stationary activity bump emerges even in the absence of external cues or shifting signals when the initial condition (*u*,*q*) takes the same. The stationary bump can manifest at any position. (A) The experimental schematic without inputs. (B) (Left) The space-time plot of the excitatory population. Different initial values can result the bump to persist at various positions. (Right) The profile of activity bumps corresponding to the left plot at *t* = 1000 ms. (C) When *α* = 400, the activity bump is unstable and quickly dissipates. (D) When *α* ≤ 40, the ring attractor network consistently sustains itself in the dark, where ‘p’ denotes persistence. As *α* gradually increases, the actual bump profile decays until it becomes flat. The duration of the activity bump exhibits a negative nonlinear relationship with the parameter *α*.

To validate the model's capacity to preserve original information through sustained activities in the absence of inputs, we set the shifting signal (e.g., self-motion input in the HD system) and external cue signal (e.g., visual input in the HD system) for the excitatory population to zero. This is achieved by setting *ω* to zero (refer to Equation (5)) and *I* to zero (refer to Equation (1)). Under these conditions, the network rapidly settles into a stationary bump, representing persistent activity in the head direction neurons. Due to the rotational symmetry characteristic of the ring attractor network, the stationary bump is equally likely to manifest in any direction, thereby capable of representing an arbitrary head direction. The upper and lower panels of Fig. 3(B) illustrate stationary bumps maintained at x=π/2 and x=π, respectively. These results (Fig. 3 and Supplementary Video 1) are consistent with those observed in a biological experiment [8] where the activity of E-PG neurons persisted even in the absence of inputs.

Being unique components added to the traditional ring attractor, synaptic processing may play a pivotal role in shaping activity-maintaining abilities. To evaluate this hypothesis, we systematically vary the recovery rate (*α* in Equation (2)) of available presynaptic resources and assess its impact on the maintenance of neural activity. The results, as presented in Fig. 3(D) demonstrate that when the resource recovery rate is sufficiently rapid (α≤40 ms), activity can be maintained permanently. Conversely, when *α* exceeds 40 (noting that *α* appears in the denominator in Equation (2), so a lower *α* corresponds to faster recovery), the duration of sustainable activity decreases. This decrease in sustainable activity results from alterations in *α*, which induce a shift to a different equilibrium state. This conclusion is reminiscent of findings in [23], where connection strength was found to affect activity maintenance ability.

#### 2.1.2 Traveling activity bump according to the shifting inputs

**Figure 4:**
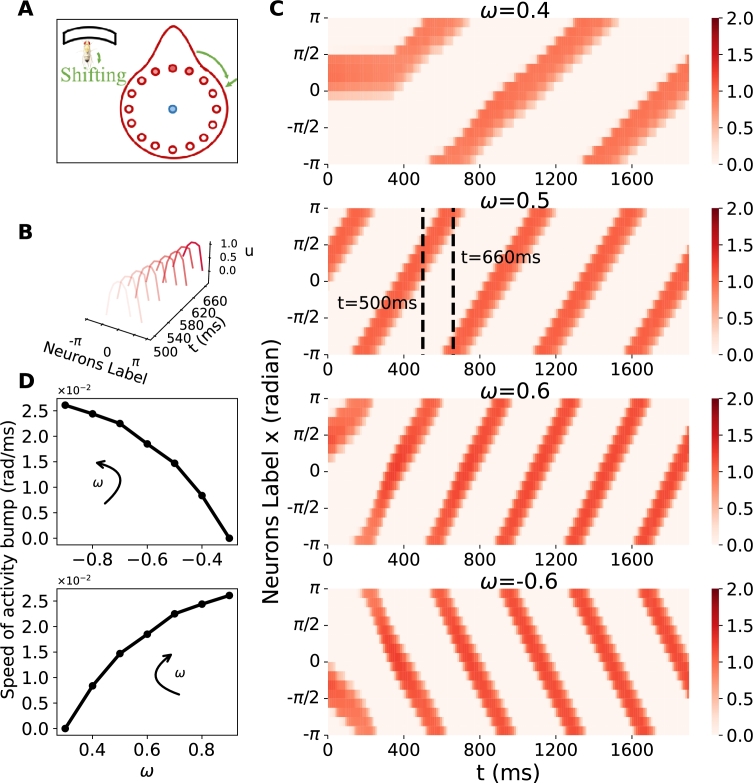
**Traveling activity bump with various shifting inputs.** A traveling activity bump tracks shifting signals when *I*^*ext*^ = 0, *ω* ≠ 0 and the initial condition is set as a single peak. (A) The experimental schematic with shifting input. The shifting input is represented in green. (B) The profile of a traveling bump with *ω* = 0.5, corresponding to the (2nd figure in C). The abscissa denotes neurons labeled by their preferred directions; while the ordinate represents time (500-660 ms); the vertical coordinates depict synaptic currents. (C) The space-time plot of the excitatory population. The ring attractor network shows the spatially tuned pattern propagates at various constant speeds for *ω* = 0.4,0.5,0.6,−0.6. Among these, the fastest propagation occurs at *ω* = 0.6, whereas propagation in the opposite direction is represented at *ω* = −0.6. (D) (Top) When rotating counterclockwise, the speed of the traveling activity bump is negatively correlated with shifting input. (Bottom) Conversely, when rotating clockwise, the activity bump travels in the opposite direction, and the speed is positively correlated with the shifting input. As *ω* increases, the traveling speed also increases and tends to saturate.

To evaluate the model's ability to integrate shifting inputs and dynamically update activations without external cues, we set the external input $I^{ext}=0$ (refer to Equation (1)) and introduce a shifting signal ω≠0 (refer to Equation (5)). Simulation results show that the activation bump no longer stays stationary but travels with a constant velocity (Fig. 4(B)).

This phenomenon emerges due to the asymmetry in synaptic currents induced by the shifting inputs. Specifically, in biological experiments of [8], see Fig. 4(A)), compass neurons can update the orientation representation by integrating self-motion cues when *Drosophila* walks in darkness, in the absence of visual input. In these experiments, the traveling speed of the activity bump increases proportionally with the increased strength of the input signal, indicating the model's ability to effectively integrate various shifting signals (refer to Fig. 4(C)).

We systematically vary *ω* across a range of values to investigate the relationship between the traveling speed of the bump and the strength of the shifting signal. The results, depicted in Fig. 4(D), show a clear positive correlation between the activity bump speed and the strength of the shifting signal. This correlation highlights that the bump-traveling speed increases at a diminishing rate as the shifting signal maintains a constant ascent.

This nonlinear relationship underscores the constrained capability of the proposed network in tracking shifting cues. However, this nonlinearity is explicable, as limitations in neural processing suggest that the system cannot track arbitrarily fast speeds. Moreover, there is indirect evidence pointing towards the underestimation of self-motion velocity [45]. Therefore, although this phenomenon has yet been confirmed through empirical biological experimentation, it is plausible to anticipate analogous performance within the neural systems of actual organisms.

### 2.2 Ring attractor as an cues integrator

Physiological investigations have reported cue integration behaviors within the rat's HD system, while behavioral studies have revealed insects' remarkable ability to optimally integrate conflict cues during navigational tasks. For instance, when rats are presented with two small conflict visual cues, HD cells demonstrate Bayesian integration [3]. Conversely, when insects are confronted with two large conflict visual cues, such as two stripes displayed on an LED screen, the activation patterns of their compass neurons exhibit a winner-take-all (WTA) mechanism, where the neurons align with the position indicated by one of the stripes [8]. Modeling studies using ring attractor networks have also explained these characteristics [30], [33], [32].

To investigate whether the proposed ring attractor network with synaptic processing could manifest these cue integration properties, we conduct simulations by injecting different numbers of external cue inputs (two or more) with varying conflicts.

#### 2.2.1 Switching between weighted averaging integration and winner-take-all with two conflict cues

**Figure 5:**
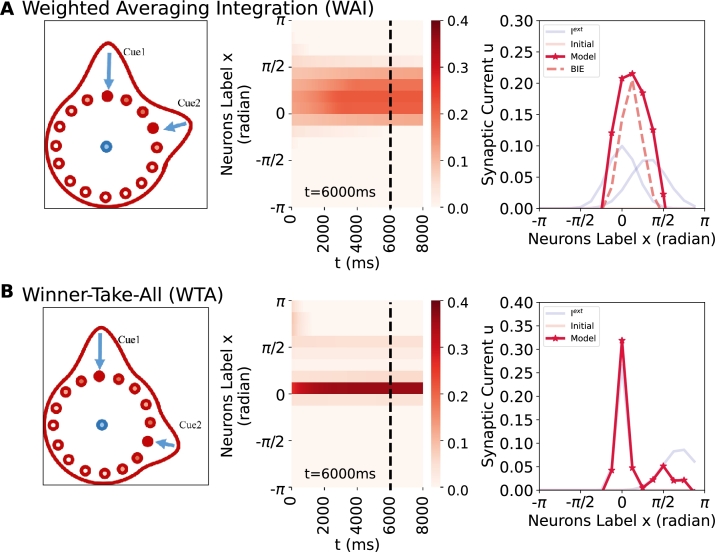
**Weighted averaging integration (WAI) and winner-take-all (WTA) with two external conflict cues in a ring attractor network.** The phenomena of WAI and WTA occur under conditions where *I*^*ext*^ ≠ 0, *ω* = 0 and the initial condition (*u*,*q*) is set to 0. The external cue input is (e.g. vision, olfactory, wind direction, tactile etc.) represented in blue, the Bayesian integration estimate (BIE) is represented in dashed, and the model's output is represented in red. The parameter values can be found in Table 3(a)-(b). (Left) The experimental schematic. (Middle) The space-time plot. (Right) The profile plot. (A) In the presence of two small conflict external cue inputs, the model shows weighted cue averaging integration, maintains a stable response. (B) In the presence of two large conflict external cue inputs (weak and strong), the model exhibits a WTA response and remains stable. The case of 128 neurons can be seen in Fig. S6.

Firstly, we assess the integration performance of the network when presented with two conflict cues. Our analysis is conducted under two conditions: different cues with small conflicts (Table 3(a)); different cues with large conflicts where Cue1 is stronger than Cue2 (Table 3(b)). Fig. 5 shows the cues and the responses of the ring attractor network, represented by blues and red respectively. In Fig. 5(A), Cue1 is oriented towards x=0 rad, while Cue2 is oriented towards x=1 rad. Notably, model's response peak around x=0.39 rad, demonstrating a weighted cue averaging integration that aligns more closely with Bayesian integration (BI) than with average-sum integration (Fig. 15). The neuron with the highest activity remains stable over time, maintaining a response profile consistent with that observed at t=6000 ms (refer to Fig. 5(A, middle)). Fig. 5(B) illustrates a winner-take-all phenomenon when two large conflict cues are presented. In this condition, the neuron with the highest activity is located approximately at the position of the strongest cue. Similar to the stable state in the previous case, this neuron's activity also remains stable over time. These results are similar with the behavioral studies of integrating multiple cues [13], [14], [15], [16].

**Figure 6:**
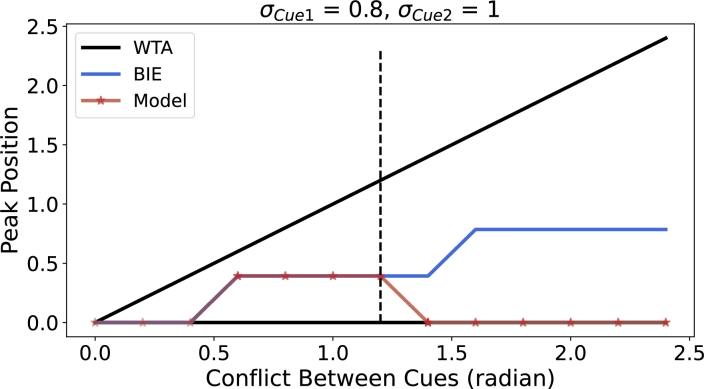
**Network performance of weighted cue averaging integration (WAI) and winner-take-all (WTA) with increasing cue conflict.** Cue1 (*μ*_*Cue*1_ = 0) is consistently presented at a fixed location, while Cue2 is presented at increasing distances. The WTA prediction is represented in black, the BIE prediction is represented in blue, and the response of the ring attractor network is represented in red. When *σ*_*Cue*1_ = 0.8, *σ*_*Cue*2_ = 1, the model exhibits distinct phases of integration: weighted cue averaging integration WAI (0-1.2) and WTA (1.2-2.4). The network performance for the case of 128 neurons is illustrated in Fig. 16.

In order to delineate the critical conditions for the proposed network's transition from weighted cue averaging integration (WAI) to winner-take-all (WTA), we investigate the network's performance as the conflict between two external cue inputs increased (Fig. 6 and Supplementary Video 2). When cues with different variance (i.e., $\sigma_{Cue1}$ = 0.8 and $\sigma_{Cue2}$ = 1) are employed, the model output exhibits a shift from weighted cue averaging integration to WTA when conflict exceeds approximately 1.4 (Fig. 6). Notably, the system performs integration within the conflict range of 0 to 0.4, despite Fig. 6 exhibiting characteristics resembling WTA behavior. This occurs due to the constrained neuronal population, where minor differences within the 0 to 0.4 range are not adequately resolved, resulting in a stepped appearance in the curve. To provide further elucidation, we added supplementary experiments depicting the responses of 128 neurons, as shown in Fig. 16, Fig. S6. As illustrated in Fig. 16, increasing the number of neurons to 128 allows the BIE to approximate a continuous sloping straight line with a consistent gradient.

In a word, the feature of effectively integrating two cues, characterized by optimal integration with relatively small conflicts and competition with relatively large conflicts, which reveals a property that aids in explaining the diversity of animals' cue integration behavior for various sensory inputs [3], [15], [31]. This insight can also provide guidance for designing sensor fusion algorithms [46], [47].

#### 2.2.2 Integrating more than two cues in the similar manner

Given the evidence indicating that animals perceive their living surroundings through multimodal sensory information, encompassing not only vision but also olfactory, tactile, and mechanosensory etc. Thus, it is imperative to understand how neural systems integrate more than two sensory inputs. For instance, the heading direction system of insect has been demonstrated to incorporate vision, olfactory and so on [7], [9], [10], [11], [12]. However, there is a paucity of modeling studies addressing the integration of more than two sensory cues. To bridge this gap, this section examines the performance of the proposed ring attractor network when it receives more than two external cue inputs.

**Figure 7:**
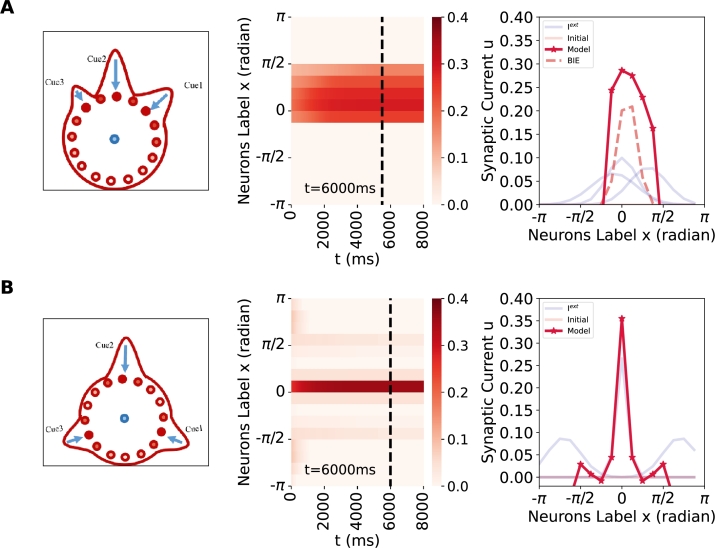
**Weighted cue averaging integration and winner-take-all (WTA) with three external conflict cues.** The phenomena of weighted cue averaging integration and winner-take-all behavior when *I*^*ext*^ ≠ 0, *ω* = 0, and the initial condition is set to 0. The external cue input is represented in blue, the Bayesian integration estimate (BIE) is represented in dashed, and the model's output is represented in red. Parameter values can refer to Table 3(c)-(d). (Left) The experimental schematic with three different conflict internal cue inputs. (Middle) The space-time plot displays the excitatory population dynamics. (Right) The profile plot. (A) In the presence of 3 small conflict external cue inputs (identical), the model's response shows weighted cue averaging integration, and remains stable. (B) In the presence of 3 large conflict external cue inputs (strong, weak), the model demonstrates a stable WTA phenomenon.

Firstly, in examining the case of three external cue inputs, we systematically vary the certainty and conflict levels of the inputs, as these factors are critical in determining the model's cue integration performance. Numerous simulations are conducted with various combinations of input settings, revealing two typical performances. 1) In Fig. 7(A) and Table 3(c), when the three cues possess different levels of certainty and the conflicts among Cue1&Cue2&Cue3 are relatively *small*, the network tends to integrate the cues by weighted averaging, similar to a Bayesian integration estimate. Fig. 7(A, right) shows the proximity between the Bayesian integration estimate (BIE) and model output. 2) In Fig. 7(B) and Table 3(d), when the three cues exhibit varying degrees of certainty and conflicts between Cue1&Cue2 and between Cue2&Cue3, are relatively *large*, the network tends to favor the position associated with the most certain cue, resulting in a winner-take-all phenomenon. It is crucial to comprehend how the ring attractor network resolves the conflicts and whether it continues to prioritize the most certain cue. The ring attractor network addresses such conflicts by exhibiting a preference for the weighted averaging of cues with smaller conflicts. It then assesses the certainty and conflict of the integrated cue against the remaining cue, revealing that the cue with the highest certainty exerts the predominant influence (i.e. WTA).

**Figure 8:**
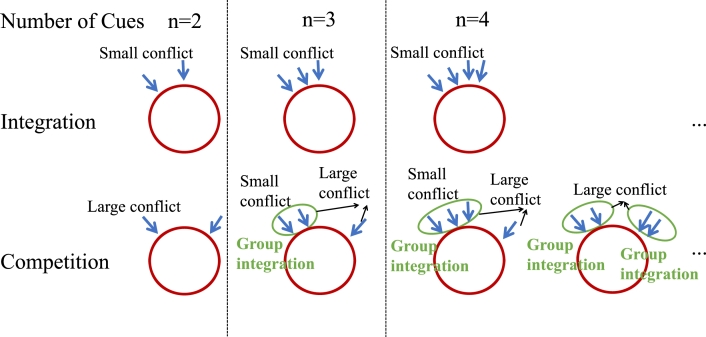
**Integrating multiple cues using a grouping approach.** The blue arrows indicate external cue inputs. The intrinsic mechanism of the ring attractor network governs its tendency to organize these cues based on the strength of their conflicts. Initially, it integrates cues characterized by low conflict levels and subsequently addresses the competition among the grouped cues.

Secondly, to deal an arbitrary number of external cue inputs (i.e., cases with more than three cues), we propose a grouping approach inspired by the performance of the model and the principles of combination and WTA concept [48]. Specifically, as shown in Fig. 8, different cue inputs can be grouped according to the degree of conflict among them. Then, cue inputs within the same group are optimally integrated via a weighted averaging method. In other words, cues with small conflicts can be regarded as a single cue, with an estimation derived from the weighted averaging integration of those cues. Following this grouping process, the number of inputs is reduced, simplifying the situations to cases with two or three cues. These simulations provide a demonstration of the model's response to combinations of cues with varying levels of conflict, providing insights into the processing of multiple cues within the ring attractor network across diverse sensory domains. Although the original real-world experiment did not involve the use of multiple cues, the model's predictive capacity allows for informed conjecture regarding the behavior of animals in such contexts.

### 2.3 Ring attractor integrates moving external input and shifting input

In the previous sections, the simulations exclusively involved static external inputs. However, evidences indicate that animals can track moving external cues [8], [9], [49]. This ability proves valuable as it allows animals to dynamically update their internal representation of a specific environmental variable, facilitating immediate responses. In [8]'s *Drosophila* experiment, the influence of the shifting input on the capacity of compass neurons to track a moving external visual cue is also investigated. Nevertheless, these results have not yet been explained by any computation model. In this section, we first assess whether the proposed network could dynamically track an moving external cue at different speed. Subsequently, we aim to replicate the results of the *Drosophila* experiments to ascertain whether the proposed model effectively accounts for the reported biological behaviors.

#### 2.3.1 Tracking the moving external cue input

**Figure 9:**
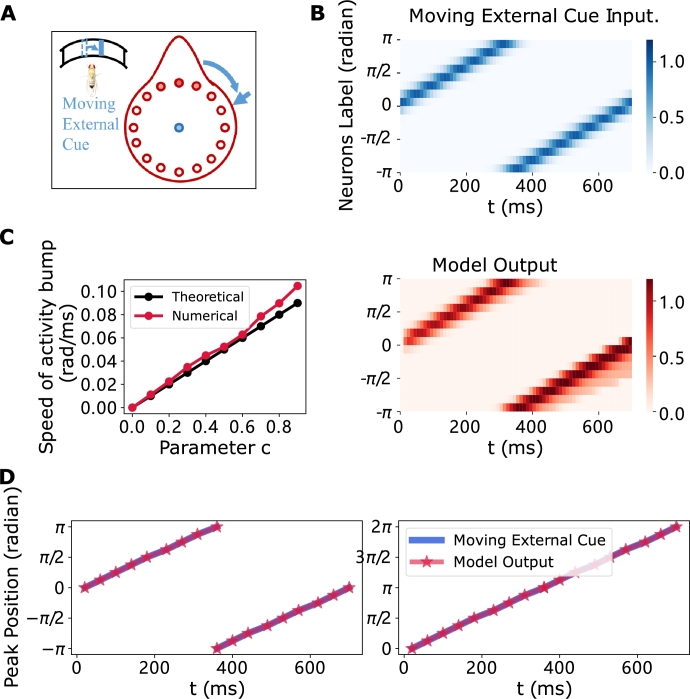
**Traveling activity bump with moving external cue inputs.** Parameters are set at *c* = 0.09, *ω* = 0 and an initial condition characterized by a single peak. The moving external cue input is represented in blue, and the model's output is represented in red. (A) The experimental schematic with moving external cue input. (B) The space-time plot of the moving external cue input (Top) and model output (Bottom). (C) As parameter *c* increases, the speed of activity bump also increases. Theoretically, this relationship is nearly linear (depicted by the black lines), but numerical results (indicated by the red lines) reveal slight deviations (due to the lower number of neurons). (D) Peak position plot. The model exhibits precisely tracks the moving external cue input. It is noteworthy that the real output is represented on the left plot, while the right plot unfolds the periodic behavior (ordinate axis) for ease of observation. The right depiction in the interval (*π*,2*π*) mirrors that of (−*π*,0) within the left plot. The “Peak Position” graphs below follow the format of the right plot in several instances.

To assess the capability of the proposed ring attractor network's in tracking moving external cues, we modify the external cue input from $I^{ext}(x)$ to $I^{ext}(x,t)=I^{ext}(x-ct)$, where *c* represents the moving speed of external cue. While maintaining the bell-shaped profile of the input, consistent with previous simulations, we allow the peak position to travel at a specific angular speed (rad/ms), denoted by *c* over time. In this system, the temporal evolution constant is denoted as *τ* = 1 (10 ms). For this specific time constant, the propagation speed of a moving external cue is $\omega_{I^{ext}}=c/\tau$. The model's output, depicted in Fig. 9(B), suggests that the model effectively tracks the moving external cues. To further investigate the model's capacity to track cues with varying speeds under condition of consistent certainty, we adjust the parameter *c* to modulate the moving speed of the external cue. Subsequently, we calculate the corresponding traveling speed of the network's activation bump. As depicted in Fig. 9(D), our findings reveal that the traveling speed of the input and the output (the activation bump) are nearly identical. Consequently, we can deduce that the network consistently maintains its performance in tracking moving external cues, regardless of the input's traveling speed under specified conditions of certainty.

#### 2.3.2 Preferring moving external cue to the shifting input

**Figure 10:**
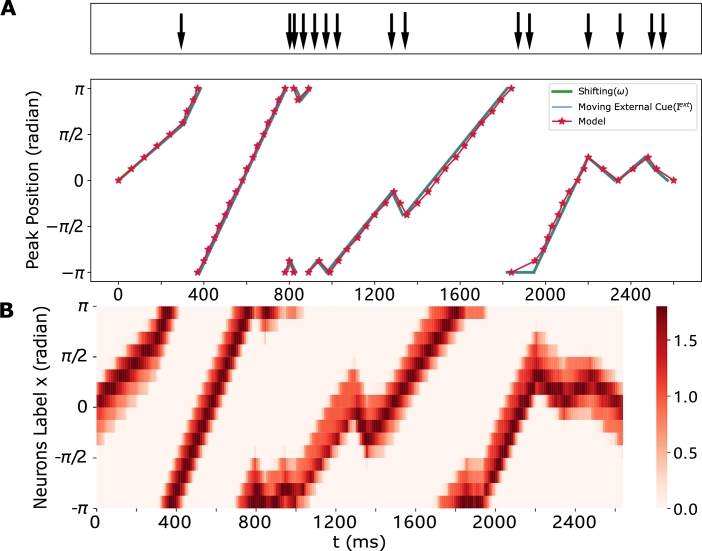
**Traveling activity bump effectively track diverse external cue input and shifting input under normal gain conditions.** The external cue input is represented in blue, the shifting input is represented in green, and the model's output is represented in red. Black arrows indicate changes in direction or speed. (A) The peak position propagates at a constant speed over time. The ring attractor model accurately tracks both the moving external cue input and shifting input. (B) The space-time plot displays the output of the ring attractor network, capturing the response to both types of inputs.

The tracking capacity of the proposed model has been verified in the previous subsection under conditions where no shifting signal input was present. However, in the biological experiment of [8], [50], animals also receive simultaneous self-motion cues while tracking a moving bar on the screen via a closed-loop setup. To replicate this property, we set the angular speed of the traveling external cue as the product of the shifting signal strength and the gain, i.e., $\omega_{I^{ext}}=Gain\cdot \omega_{Shifting}$. We change the value of the Gain to mimic the low-, normal- and high-gain experimental settings reported by [8]. In each simulation, both external cues and shifting cues are injected simultaneously, the model's output with a normal Gain is illustrated in Fig. 10, Fig. S1 and Supplementary Video 3. In this scenario, the model consistently tracks both inputs, despite of changing in their velocity and direction. Furthermore, the model's output with varying Gain values is presented in Fig. S2, Fig. S3 and Supplementary Video 3, respectively. Our findings indicate that the model's output is dominated by the external cue input, irrespective of the value of Gain. This observation aligns with results obtained in the actual biological experiment.

**Figure 11:**
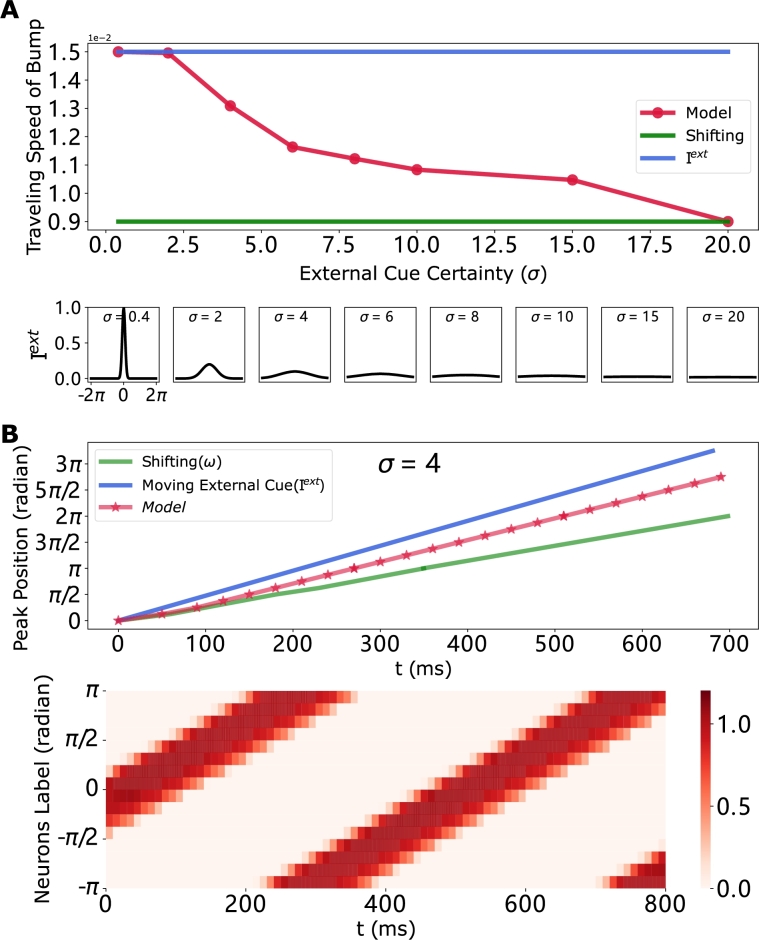
**The traveling activity exhibits an integration of shifting and external cue inputs, which correlates with a reduction in the certainty of the external cues.** Parameters are set to *c* = 0.15 and *ω* = 0.43, and the initial condition is set to consistent. The color-coding used here align with the conventions detailed in Fig. 10. (A, Top) The plot illustrates the traveling speed of the bump as it moves away from the external cue, with decreasing certainty of the cue. (A, Bottom) The plot depicts the moving external cue signal with varying levels of certainty. (B) The peak position plot and space-time plot of the ring attractor network are presented for *σ* = 4. Notably, the periodic behavior is unfolded and visualized in the (B, upper) panel, similar with Fig. 9(D, Right). In this scenario, owing to the low certainty of the moving external cue signal, the model does not precisely track the external signal but instead integrates it with the shifting signal.

According to our previous experiments, it is evident that integration performance is significantly influenced by the certainty of the input signal. Therefore, to investigate if the model exhibits cue-integrating behavior under different circumstances, we gradually decrease the certainty of the moving external cue input (Fig. 11(A)), while keeping the shifting signal constant. The results suggest that the proposed ring attractor is capable of integrating both the moving external cue input and the shifting signal even when they are conflicting, similar to the behavior described in Section 2.2.1. The findings indicate that the dominant impact of external cue input on the shifting signal, as posited in [8], represents a special scenario where the external cue input is sufficiently strong. Based on our observations, we hypothesize that in the experiment conducted by Seelig et al. [8], if the bar displayed on the screen becomes dim or less salient, the compass neurons will not track the visual cue but will instead adjust their activation to the position indicated by the shifting input. Furthermore, when the external cue input diminishes to zero, i.e., the agent is in the darkness, the model updates the activation merely based on the shifting signal (Fig. 4). This deduction further proves the feasibility of our results.

The previous section (Section 2.3.1) has demonstrated the proposed model's capability to accurately track moving external cues at arbitrary speeds under specific certainty conditions (σ<2.5). However, we aim to investigate whether this tracking ability is preserved when a shifting signal input is injected simultaneously. To explore this, we varied the moving speed of the external cues by adjusting the Gain while keeping *ω* constant. Notably, when the speed exceeds 0.018 rad/ms ($1.032^{\circ }/ms$, i.e., Gain>2), the model fails to closely follow the external cues.

**Figure 12:**
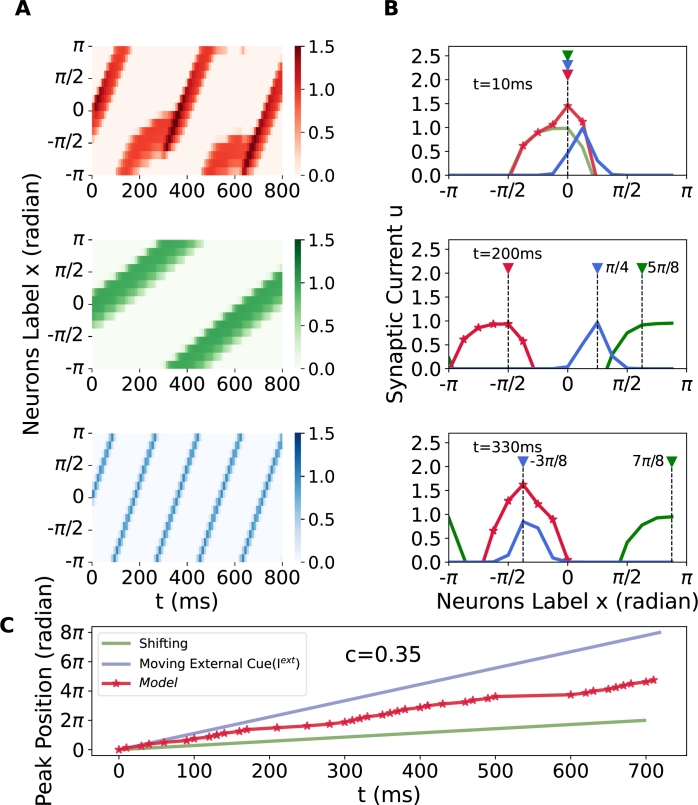
**The lag phenomenon in tracking external cue input is examined within the framework of rapidly moving external stimuli.** Parameters are set to *c* = 0.35 and *ω* = 0.43, and the initial condition is set to consistent. The color-coding and symbols used here align with the conventions detailed in Fig. 10. (A) The space-time plot illustrates the output of the ring attractor network, along with shifting input and external cue input. The speed can be derived from the slope of space-time plot. (B) Profiles of the traveling bump at t = 10, 200, 330 ms. Notably, in (B, bottom), despite the overlap in peak positions between the output and external cue input, a disparity exists in the actual phase. (C) The peak position of the ring attractor network. Notably, the periodic behavior is unfolded and visualized, similar with Fig. 9(D, Right). In this scenario, the model initially tracks the moving external cue input, and subsequently switches to the shifting speed. This transition in tracking behavior persists throughout the observed period.

Instead, it oscillates between the two signals, preceding the shifting signal while lagging behind the external cue signal (Fig. 12(C)). In this situation, the model exhibits continuous switching behavior throughout the observation period. Initially, it tracks the moving external cue, but subsequently aligns with the shifting speed within 170-280 ms and continues this pattern. The phase delay evolves over time, and as anticipated, the model's response may realign with either the external cue input or the shifting input after several cycles. Therefore, we can conclude that under the specific certainty conditions (σ<2.5), the proposed ring attractor network with synaptic processing can perfectly track external cues moving at arbitrary speeds without shifting input. However, when a shifting signal is introduced simultaneously, the model exhibits a tracking switch. This switch is caused by the observed decline in the network's response activation as the moving speed of the external cue increases. The decreased response impacts the dominance of the moving external cue, resulting in the cue integration performance described above.

## 3 Conclusion and discussion

A ring attractor network incorporating adaptive synaptic processing has been proposed, which successfully replicates significant dynamics observed in biological simulations. In summary, the model demonstrates the ability to maintain its activity bump even in the absence of inputs [43], [44], adjust its activity bump in response to shifting inputs [8], [1], and integrate cues from various sources through weighted cue averaging [13], [15], [16]. Additionally, our research addresses previously unexplored neural dynamics within the animal's HD system. These include: 1) the critical conditions of transition in a ring attractor network from cue integration performance to a WTA mechanism [3], [17], [9]; 2) the impacts of shifting inputs on compass neurons' ability to track moving external cues [8], [9], etc. We provide biologists with testable predictions derived from systematic simulations, aimed at deepening the understanding of attractor dynamics. For instance, we evaluate the performance of the ring attractor network when exposed to multiple external cue inputs, integrating them akin to situations with two cues. Additionally, we investigate potential outcomes when the model combines conflicting moving external cue input with shifting input. Future biological studies based on these predictions could yield insights into the substrates and mechanisms underlying complex attractor dynamics in the animal brain across diverse sensory domains. Overall, this study sheds light on the potential utility of the ring attractor network with adaptive synaptic processing in modeling neural systems and understanding complex neural phenomena.

### 3.1 Linking to different types of bio-plausible ring attractor networks

In the model based on the now-known architecture and neural connections [18], [19], [30], the interactions of neural circuits were considered to integrate angular velocity signals within heading circuits. Nevertheless, our proposed framework introduces a more general fundamental neural network architecture relying on a single ring attractor network. Any neural network meets this form can be a specific implementation of our model. For example, excitatory neurons EP-G, P-EG, P-EN and inhibitory neuron Pintr in Protocerebral Bridge-Ellipsoid Body of Drosophila can be potential candidates corresponding to the neurons in our model [19]. Thus, our model can be applied to explain neural dynamics in both insects and rodents without hard coorelation to a specific neural network. The architecture of a single ring attractor network offers the distinct advantage of simplicity and effectiveness in simulating neural computations within the brain, while accurately capturing key features. Additionally, for the purpose of elucidating more intricate phenomena, we may expand the model by incorporating additional ring attractors, thus adapting it to accommodate more intricate functions.

### 3.2 Bistable perception under ambiguous cues

The slow recovery speed (*α* = 400 ms) of available presynaptic resources gives rise to a notable phenomenon of bistable perception. This phenomenon is characterized by the spontaneous alternation or rivalry of two perceptual interpretations in response to stimuli with sensory ambiguity. Evidence of bistable perception has been observed in the insect brain, particularly in the central complex (CX) [8], [53], [9].

**Figure 13:**
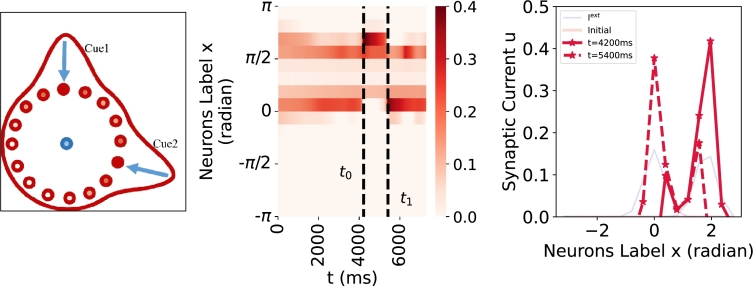
**Bistable perception with ambiguous cues** (Left) The experimental schematic with ambiguous cues. (Middle) The space-time plot. (Right) The profile plot. The moving external cue input is represented in blue, and the model's output is represented in red. In the presence of two ambiguous cues (*μ*_1_ = 0, *μ*_2_ = 1.8 and *σ*_1_ = *σ*_2_ = 0.5), the ring attractor network shows switching between Cue1 and Cue2, the location of the maximum response undergoes dynamic changes, as illustrated in the profiles on the right corresponding to t=4200 ms and t=5400 ms.

As depicted in Fig. 13, a switching phenomenon becomes evident when two ambiguous cues are presented. The response of the ring attractor system ceases to remain stable, the peak response position exhibits temporal variations. At t=4200 ms, the model exhibits maximal responsiveness to Cue2, while at t=5400 ms, maximal responsiveness is observed towards Cue1. This behavior not only highlights a remarkable feature of the network but also aligns with physiological studies of [8].

**Figure 14:**
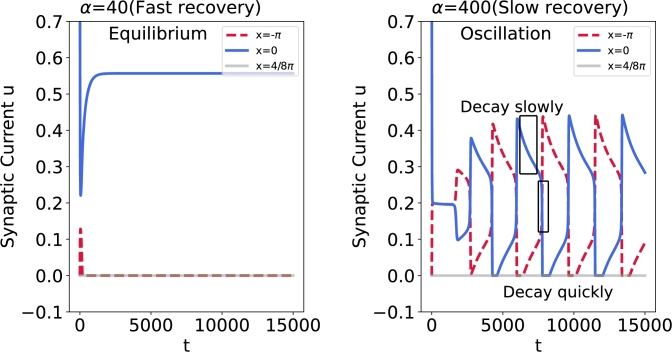
**Time history diagram of synaptic current performance (Oscillation and equilibrium) with different recovery speed.** The time history diagram illustrates the performance of synaptic currents under varying recovery speeds, showcasing oscillation and equilibrium behaviors. The red dashed line represents the neuron located at *x* = −*π*, the solid blue line denotes neuron located at *x* = 0, and the gray line denotes neuron located at *x* = 4/8*π* and neurons not shown in the figure coincide with the gray line. (Left) When *α* = 40 ms, neurons rapidly converge to different distinct equilibria, exhibiting WTA phenomena. (Right) When *α* = 400 ms, the responses of two neurons cease to remain stable, instead, they converge to two oscillation attractors respectively, demonstrating continuous switching phenomena. Specifically, the neuron at *x* = 0 experiences slow decay followed by quickly decay, while the neuron at *x* = −*π* undergoes the slow increase followed by rapid increase.

Different from the traditional ring attractor networks, our model incorporates a reciprocal inhibition architecture, i.e. synaptic depression, which is widely used to describe bistable perception in general. Within this framework, the system generates a dominant side and a competing side. The switch in dominance between the two sides occurs through a slow negative feedback process that decreases the activity of the dominant side, allowing the suppressed population to become active (Fig. 14). In summary, bistable perception can be accommodated within the ring attractor framework. Although we just have demonstrated a simple instance (Fig. 13), this model can be extended to more complex situations. For instance, the combination of the attractor and stochastic integration has been employed to explain more dynamical possibilities [54], [55].

### 3.3 Effect of parameters on proposed model

The integration capabilities of the ring attractor network, particularly its weighted cue averaging mechanism, are of significant research interest. Analytical solutions to equations involving convolutional terms are notoriously difficult to derive. Zhang et al. selected a specific convolutional term to derive an analytic solution, which showcasing the Bayesian optimality of the ring attractor network [40]. In contrast, our study aims to demonstrate Bayesian-like optimality through numerical simulations.

**Figure 15:**
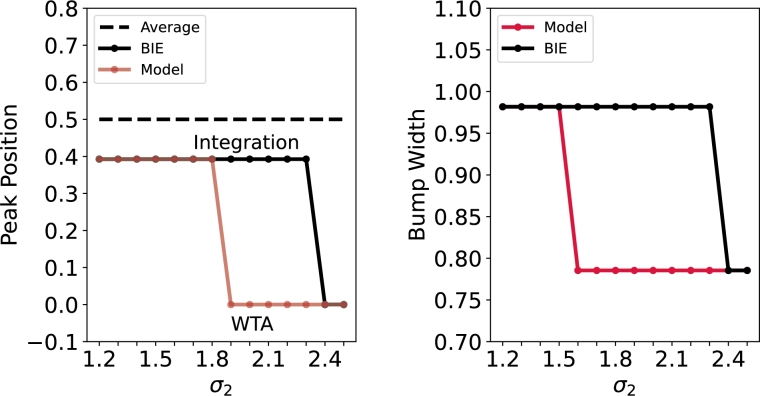
**The effect of***σ*_2_**on ring attractor network's weighted cue averaging integration.** The parameters are set as follows: *μ*_1_ = 0, *μ*_2_ = 1, and *σ*_1_ = 1.2. (Left) When *σ*_2_ < 1.8, the peak position of the BIE and the model remains consistent. But when *σ*_2_ > 1.8, the system behavior transition from Integration to WTA. Nevertheless, it does not exhibit an average trend. (Right) The bump width is defined in relation to the distance between the two points where *u* = 0 intersects the bump curve. The bump width of the model enters a distinct state (*σ*_2_ > 1.6) earlier than that of the BIE (*σ*_2_ > 2.4).

To verify the impact of parameters on the weighted cue averaging integration within the ring attractor network, we vary the *σ* and *α* parameters. Initially, $\sigma_{1}=\sigma_{2}$ are set to 1.2, representing a critical state where both signals hold equal confidence. Subsequently, we explore a scenario where Cue1 remains constant while Cue2 varies. As depicted in Fig. 15 (Left), the model consistently transitions from a state of weighted integration ($\sigma_{2}<1.5$) to a winner-takes-all (WTA) state ($\sigma_{2}>1.6$) earlier than the BIE (Bayesian Integration Estimate) as $\sigma_{2}$ increases. Additionally, Fig. 15 (Right) further illustrates that the bump width of the model enters a distinct state earlier than that of the BIE. This phenomenon is easily comprehensible: as the confidence in Cue2 progressively decreases, the system assigns greater weights to Cue1. At this stage, the system exhibits winner-takes-all (WTA) behavior, where Cue2 is neglected and the system responds solely to Cue1. This phenomenon can be attributed to the differential modulation of neuronal activity. High-activity neurons experience a balance of excitatory and inhibitory inputs, while low-activity neurons receive stronger inhibitory inputs. More details see Fig. S4.

**Figure 16:**
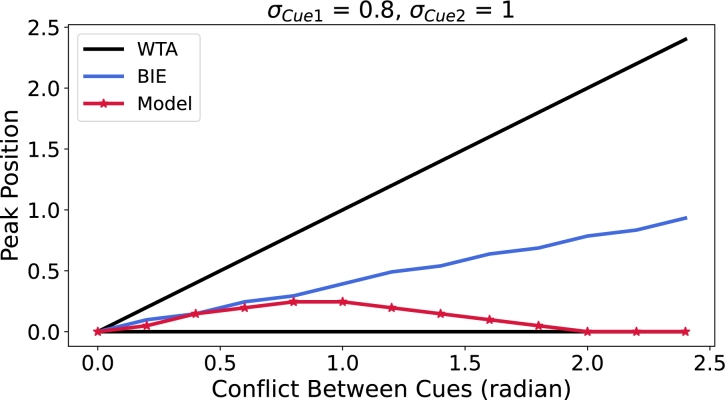
**The performance of 128 neurons in weighted cue averaging integration (WAI) and winner-take-all (WTA) with increasing cue conflict.** Cue1 (*μ*_*Cue*1_ = 0) is consistently presented at a fixed location, while Cue2 is presented at increasing distances. Additional parameters are set as *σ*_*Cue*1_ = 0.8 and *σ*_*Cue*2_ = 1. The responses of the ring attractor network are depicted by the red lines, whereas the WTA predictions are represented by the black lines and BIE predictions are represented by the blue lines.

The performance of the system is influenced by the number of neurons employed. In order to observe more detailed changes in the system on strategy switching, we set the neuron count of N=128. As illustrated in Fig. 16, with an increasing conflict of cues, the network's response approaches (though never very precisely matches) the BIE solution, but changes to WTA-like responses when cue conflict exceeds approximately 2. The profiles of weighted cue averaging integration and WTA-like mechanisms are illustrated in Fig. S6.

### 3.4 Weighted cue averaging and Bayesian integration

Traditional Bayesian integration (BI) is a probabilistic approach that integrates multiple sources of information to manage uncertainty effectively and make optimal decisions. It is widely regarded as a fundamental framework for information processing in animals. However, this paper proposes an alternative method for integrating multiple sources of information through weighted averaging. Although this method approximates the results of BI, it never matches BI exactly (see Fig. 15). Nonetheless, the proposed model offers valuable insights, particularly as it accurately reflects the location of excitatory neurons, thereby providing animals with precise directional information.

### 3.5 Building block for more complex neural circuitry

The ring attractor dynamics, proposed as cue integrators with maintainable and shiftable activation, provide a powerful and unifying conceptual framework for motor control, memory maintenance [8], [43], [44], guidance integration, decision making [13], [14], [15], [16] and spatial navigation [1], [2], [3], [4], [5]. Despite our results are primarily validated within the context of the heading direction system, they offer inspirations into neural interactions and their integration with other mechanisms. The comprehensive investigation of the model's performance enriches our understanding of the brain's ability to solve intricate problems related to intelligence. For instance, this model can be utilized as a building block, where one component retains memorized direction while the other tracks real-time direction, and the gap in direction between these two attractors can guide an organism in navigation [56], [30]. Additionally, this network can also be employed to construct place cells and grid cells [57], [58], facilitating the creation of cognitive maps through the interplay of these two cell types. The proposed model is not limited to traditional data learning [59], [60], but offers an efficient option for engineers seeking bio-inspired cues fusion algorithms [47]. Undoubtedly, the potential application areas of attractors providing exciting opportunities for future research and applications.

### 3.6 Further work

While our model adequately simulates a significant portion of the neural dynamics observed in the animal brain, it relies on simplistic input assumptions. Addressing this limitation and exploring methods to incorporate real, complex visual inputs instead of relying solely on simplistic Gaussian shapes represents a crucial avenue for future research.

## 4 Method

Our recurrent model, depicted in Fig. 2, comprises a population of excitatory neurons organized into a ring network with rotational symmetry, as documented in prior studies [61], [21], [23]. Each neuron within this network receives exogenous inputs (i.e., external cues) as well as various endogenous inputs, including shifting inputs, excitatory synaptic inputs, and inhibitory synaptic inputs. Additionally, the available presynaptic resources are regulated in synaptic processing, which influences the recovery and depletion speeds of synaptically transmitted signals. The model calculations are conducted using the fourth-order Runge-Kutta method and implemented in Python.

### 4.1 Neural model

Both the dynamics of excitatory neurons and the evolution of synaptic currents and available presynaptic resources within each neuron are described by differential equations. The details are presented as follows:

(1) $$\tau \frac{\partial u(x,t)}{\partial t}=-u(x,t)+u_{E}(x,t)+u_{L}(x,t)+u_{R}(x,t)+u_{I}(x,t)+\sum_{i}m_{i}I_{i}^{ext}(x,t),$$

(2) $$\frac{\partial q(x,t)}{\partial t}=\frac{1-q(x,t)}{\alpha }-\beta q(x,t)f(u(x,t)),$$

(3) $$\tau_{2}\frac{du^{inh}}{dt}=-u^{inh}+u_{E}^{inh}.$$

The symbol *τ* represents the time constant, we assume a time scale of τ=1(10ms). Equation (2) describes the evolution of the fraction of available presynaptic resources, denoted as q(x,t). These resources are depleted at a rate denoted by *βf* and recovered at a speed denoted by *α*. The constant *α* specifies a time scale (millisecond) and allows the factor q to recover on that time scale. When the depletion speed exceeds the recovery speed, the pool of available resources contracts, resulting in diminished synaptic activation and a slow evolution of *q*. Conversely, when the speed of resource depletion outpaces the speed of recovery, *q* evolves rapidly.

Within the model formulation, $u_{E}$, $u_{L}$, and $u_{R}$ denote excitatory inputs from itself, left neighboring neuron and right neighboring neuron, respectively. And $u_{I}$ represents the inhibitory input. These inputs can be expressed as:

(4) $$u_{E}=w_{E}f(u(x,t))q(x,t),u_{I}=\int_{r_{l}^{I}}^{r_{u}^{I}}w_{I}f(u(y,t))q(y,t)dy,u_{L}=\int_{r_{l}^{L}}^{r_{u}^{L}}g_{L}(\omega ,y)f(u(y,t))q(y,t)dy,u_{R}=\int_{r_{l}^{R}}^{r_{u}^{R}}g_{R}(\omega ,y)f(u(y,t))q(y,t)dy,u_{E}^{inh}=\int_{r_{l}^{I}}^{r_{u}^{I}}w_{E}f(u^{inh}(y,t))q(y,t)dy.$$

In the given expression, $r_{u}$ and $r_{l}$ represent the upper and lower bounds of the range, respectively. As previously discussed, $u_{L}$ and $u_{R}$ are regulated by $g_{L/R}(\omega ,y)$, which is determined by the head's angular velocity signal. When *ω* is positive, the right connection strengthens, leading to a clockwise rotation of the traveling bump. Conversely, when *ω* is negative, the left connection strengthens, resulting in a counterclockwise rotation of the traveling bump. The velocity of the traveling bump is influenced by the strength of recurrent synaptic inputs.

Inhibitory weight $w_{I}<0$ within $u_{I}$ and excitatory weight $w_{E}>0$ within $u_{E}$ are constant, while the terms $g_{L}$ and $g_{R}$ can be determined by a mirror-symmetric function:

(5) $$g_{L}=\left\{ \begin{array}{cc} & w_{L}(\omega ,y)\;\omega >0,\\ & 0\;\;\;\omega \leq 0, \end{array}\right.\;\;\;g_{R}=\left\{ \begin{array}{cc} & 0\;\;\;\omega \geq 0,\\ & w_{R}(\omega ,y)\;\omega <0, \end{array}\right.$$

The weight function *w* is distance-dependent, with its value decreasing as spatial distance increases.

As described in Equation (6), we utilize a smooth sigmoid function as the activation function. However, it's worth noting that other diverse nonlinear functions, such as the Heaviside step function, are also permissible.

(6) $$f(u)=\frac{1}{1+e^{-\lambda (u-\kappa )}},$$

In this activation function, *κ* is taken as threshold while *λ* is considered as the gain (note the distinction from Gain in the main text). The external cue input $I^{ext}$ in Equation (1), which represents various sensory inputs like vision, olfactory, wind direction, tactile etc.), can be effectively modeled using a Gaussian function (or Cosin function [34], [24]):

(7) $$I^{ext}=\frac{1}{\sigma \sqrt{2\pi }}e^{-\frac{{\left(x-\mu \right)}^{2}}{2\sigma^{2}}},$$

where parameters *m*, μ=ct and *σ* denote the magnitude, mean and variance of the Gaussian distribution, respectively. And *c* represents the moving speed of external cue input.

### 4.2 Simulation

Inspired by biology [8], continuous differential equations are discretized with the parameters N=16 and dy=π/8 (Table 2). Here, *N* represents the number of discrete points, and *dy* serves as the integral variable. The integral calculation is simplified by approximating it as an accumulative sum. In other words, the system considers 16 neurons with preference differences between adjacent neurons set at π/8, representing values such as −*π*, $-\frac{7\pi }{8}$, $-\frac{6\pi }{8}$ $-\frac{5\pi }{8}$, $-\frac{4\pi }{8}$, $-\frac{3\pi }{8}$, $-\frac{2\pi }{8}$, $-\frac{1\pi }{8}$, 0, $\frac{1\pi }{8}$, $\frac{2\pi }{8}$, $\frac{3\pi }{8}$, $\frac{4\pi }{8}$, $\frac{5\pi }{8}$, $\frac{6\pi }{8}$, $\frac{7\pi }{8}$.

**Table 2:** **Partial parameters of ring attractor network.** Other parameters are explained in the text.

Symbol	N	*β*	*w*_*E*_	*w*_*I*_	dy
Value	16	0.01	0.5	-0.5	*π*/8

In Equation (4), the variable *r* represents a specific range, defined as follows:

(8) $$\begin{array}{cc} & r_{l}^{I}=x+5dy,\\ & r_{u}^{I}=x+11dy,\\ & r_{l}^{L}=x,\\ & r_{u}^{L}=x+2dy,\\ & r_{l}^{R}=x-2dy,\\ & r_{u}^{R}=x,\\ & x\in [-\pi ,\pi ], \end{array}$$

It is important to note that the ring attractor network adheres to a periodic boundary condition, expressed as u(x+2π)=u(x).

**Figure 17:**
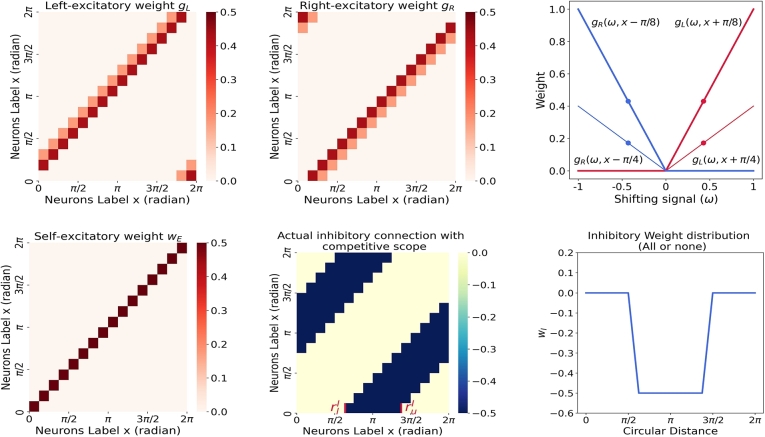
**The weight distribution of neurons in ring attractor networks.** The four plots present on the left illustrate the weight distributions for various neuronal connections, including left-excitatory (*g*_*L*_), right-excitatory (*g*_*R*_), self-excitatory (*w*_*E*_), and inhibitory (*w*_*I*_). Notably, the constant *w*_*E*_ = 0.5 in Table 2 corresponds to the entry located in the small red square within the plot. The weights associated with the left-excitatory and right-excitatory connections are modulated by a shifting input signal (*ω*). Specifically, we set *ω*_*L*_ = *ω*_*R*_ = 0.43, as depicted in the upper-right plot. The last plot represents a neuron positioned at *x* = 0, which receives inhibitory input from other neurons following an all-or-none pattern.

The weight distribution of neurons $g_{L}$ and $g_{R}$ in the ring attractor network is illustrated in Fig. 17.

To facilitate clear visualization, the system's state is plotted in a finite time (e.g., the space-time plot in Fig. S5 from 0 to 2000 ms), as it consistently stabilizes beyond this time frame. The system attains stability after 2000 ms and maintains a constant value thereafter. Given the system's convergence to an attractor, the profile at 1000 ms serves as a representative depiction of the synaptic currents.

Regarding initial values, we use fixed initial conditions. However, it is sure that the noise perturbed initial states do not impact the model's outputs (Fig. S9). The determinants of the attractor are not reliant on the initial values but rather on a combination of endogenous and exogenous inputs. Notably, the traveling wave solution always emerges from a fixed shape. When random initial values are used, the system requires more time to iterate to that shape (Fig. 4C (top): 0-400 ms).

#### 4.2.1 Simulations with shifting input

This section outlines the key parameter selection for Simulation 1 (ring attractor with maintainable and shift-able activity bump) and Simulation 3 (ring attractor integrating moving external input and shifting input). Specifically, we set the parameter *α* to a constant value of 40, while configuring the stimulus intensity ($m_{i}$, Equation (1)) to 1. The initial value of can be taken as a single peak shape. Under this configuration, the ring attractor network consistently sustains self-sustaining activity even in the absence of external cue inputs, effectively tracking inputs from both exogenous and endogenous sources.

#### 4.2.2 External cue inputs integration

For Simulation 2 (the ring attractor as an efficient sub-weighted cue averaging integrator), the critical parameters are selected as follows. We set the recovery speed α=400 (see Equation (2)) for presynaptic resources and the stimulus intensity $m_{i}=0.2$ (see Equation (1)) for simulations. The chosen value of α=400 governs synaptic depression, aligning with established experimental findings [62], [34], which typically fall within the range of 200 to 800 milliseconds. The remaining parameters are consistent with those in Table 3. The initial values were deliberately set to 0 to minimize the impact of the initial conditions.

**Table 3:** **Parameters of cues in**Fig. 5, Fig. 7.

Num.	Cue1-*μ*	Cue1-*σ*	Cue2-*μ*	Cue2-*σ*	Cue3-*μ*	Cue3-*σ*
(a)	0	0.8	1	1	None	None
(b)	0	0.5	1.8	1.8	None	None
(c)	0	0.8	1	1	-1	1.2
(d)	2.2	0.9	0	0.3	-2.2	0.9

## Data and code availability

No data was used for the research described in this paper.

All the source codes of the implementation are uploaded to Github and are available via https://github.com/yaniChen99/Synaptic-RA.git.

## CRediT authorship contribution statement

**Yani Chen:** Writing – original draft, Formal analysis, Data curation. **Lin Zhang:** Visualization, Software. **Hao Chen:** Visualization, Software. **Xuelong Sun:** Writing – review & editing, Data curation, Conceptualization. **Jigen Peng:** Funding acquisition, Conceptualization.

## Declaration of Competing Interest

The authors declare that they have no known competing financial interests or personal relationships that could have appeared to influence the work reported in this paper.

## Appendix A Supplementary material

MMC Supplementary simulation results. All supplementary results referenced in the main text are included. MMC

Video 1 Basic characteristics of ring attractor network. Video 1

Video 2 The ability of ring attractor network to integrate two external cues. Video 2

Video 3 The ability of ring attractor network to integrate external cues and shifting cues. Video 3
