# Supplementary material for: Synaptic ring attractor: A unified framework for attractor dynamics and multiple cues integration
Source: Heliyon. 2024 Aug 5;10(16):e35458. doi: 10.1016/j.heliyon.2024.e35458 (PMC11365315; doi:10.1016/j.heliyon.2024.e35458)
Supplement: MMC — Supplementary simulation results. All supplementary results referenced in the main text are included. [file mmc1.pdf]

Supplemental Figures

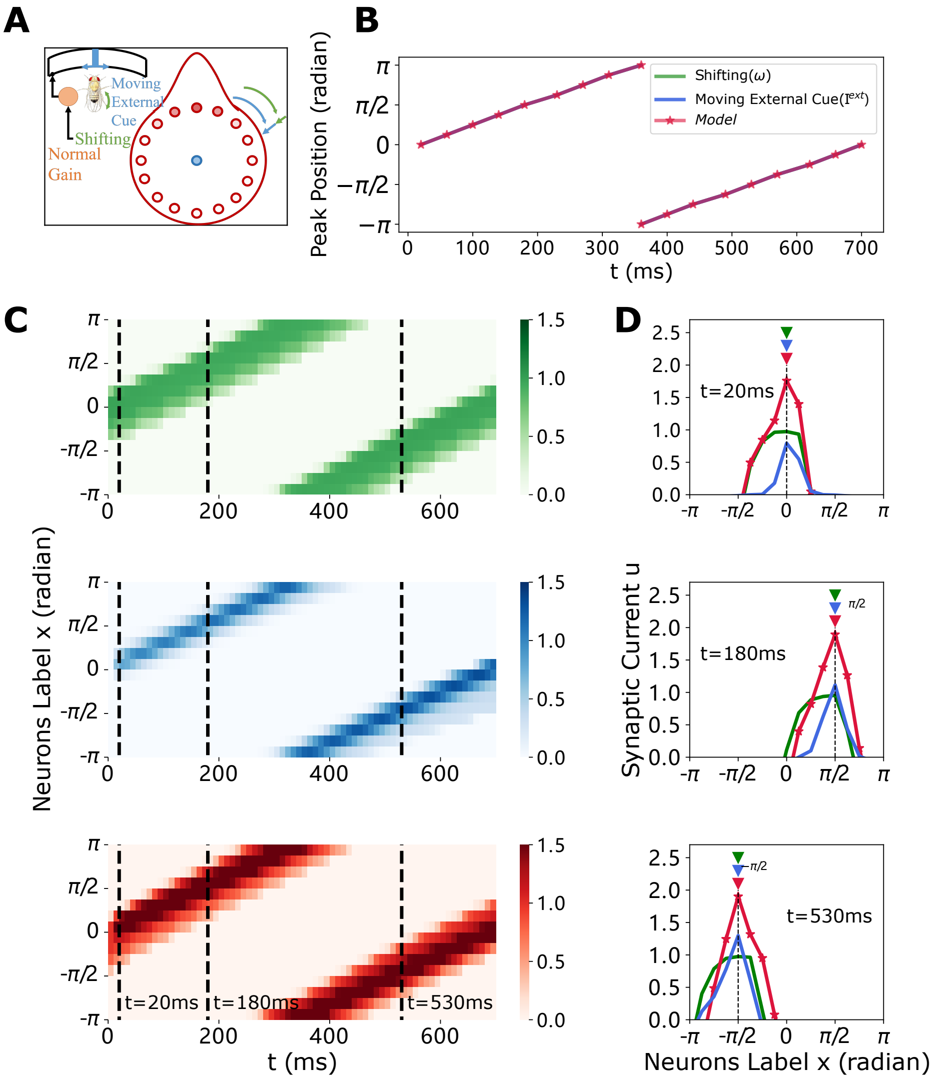

Figure S1: **Traveling activity bump tracks external cue input and shifting input in normal gain.** A traveling activity bump tracks external cue signal and shifting signal with $c = 0.09$ and $\omega = 0.43$, and the initial condition is set to consistent. The external cue input is represented in blue, the shifting input is represented in green, and the model's output is represented in red. The dashed line illustrates the relative distances of the peak positions, with small triangles indicating the corresponding horizontal coordinates. (A) The experimental schematic with two inputs in normal gain conditions. (B) The peak position propagates at a constant speed with time passing, and the ring attractor tracks the moving external cue and shifting input. (C) The space-time plot displays the shifting input, external cue input, and the output of the ring attractor network. (D) The profiles of the traveling bump are shown at t=20, 180, 530 ms, with the peak position remaining consistent across the three time points.

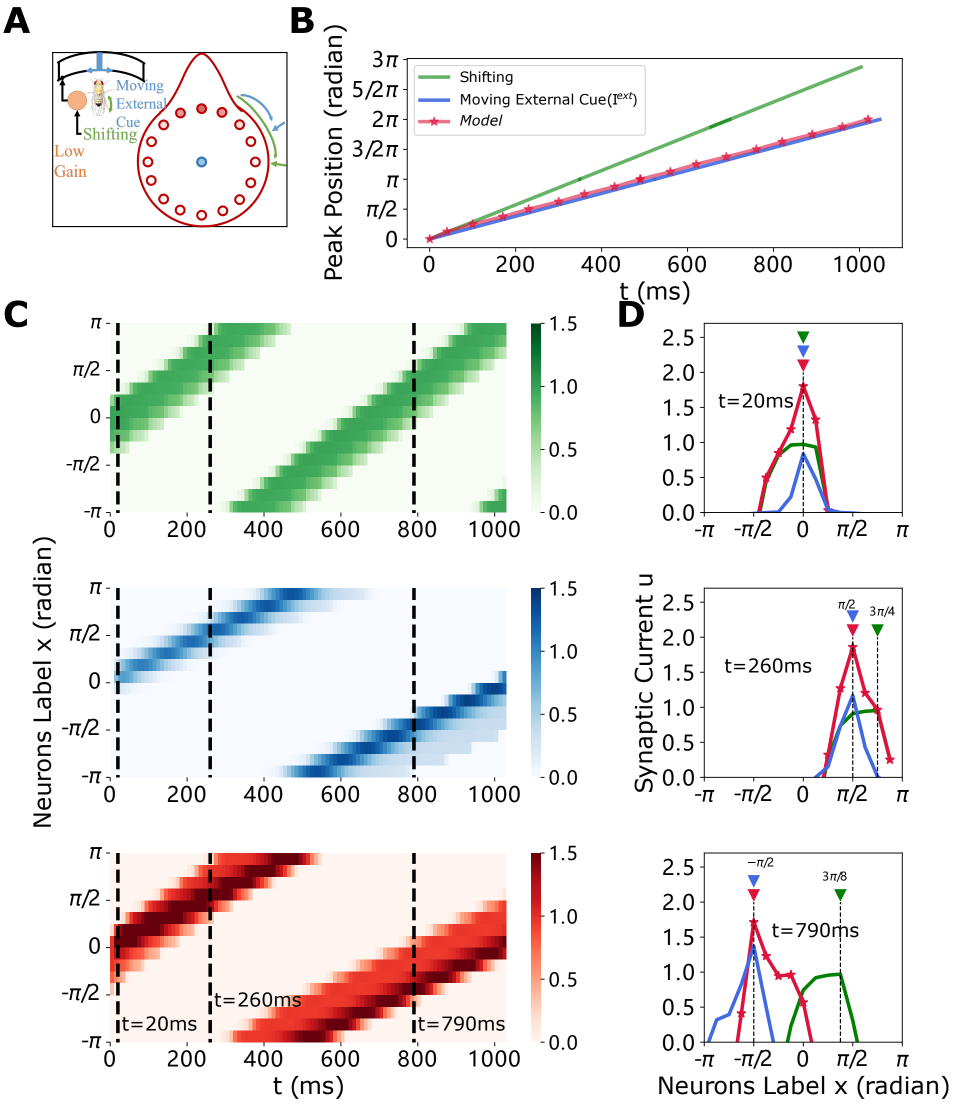

Figure S2: **Traveling activity bump with moving external cue input and shifting input in low gain.** A traveling activity bump tracks external cue signal and shifting signal with $c = 0.06$, $\omega = 0.43$, and the initial condition is set to consistent. The color-coding and symbols used here align with the conventions detailed in Fig.S1. The color-coding and symbols used here align with the conventions detailed in Fig.S1. (A) The experimental schematic with two inputs in low gain. (B) The peak position propagates at a constant speed with time passing, and ring attractor tracks the moving external cue input almost perfectly. (C) The space-time plot displays the shifting input, external cue input, and the output of the ring attractor network. (D) The profiles of the traveling bump are shown at t=20, 260, 790 ms.

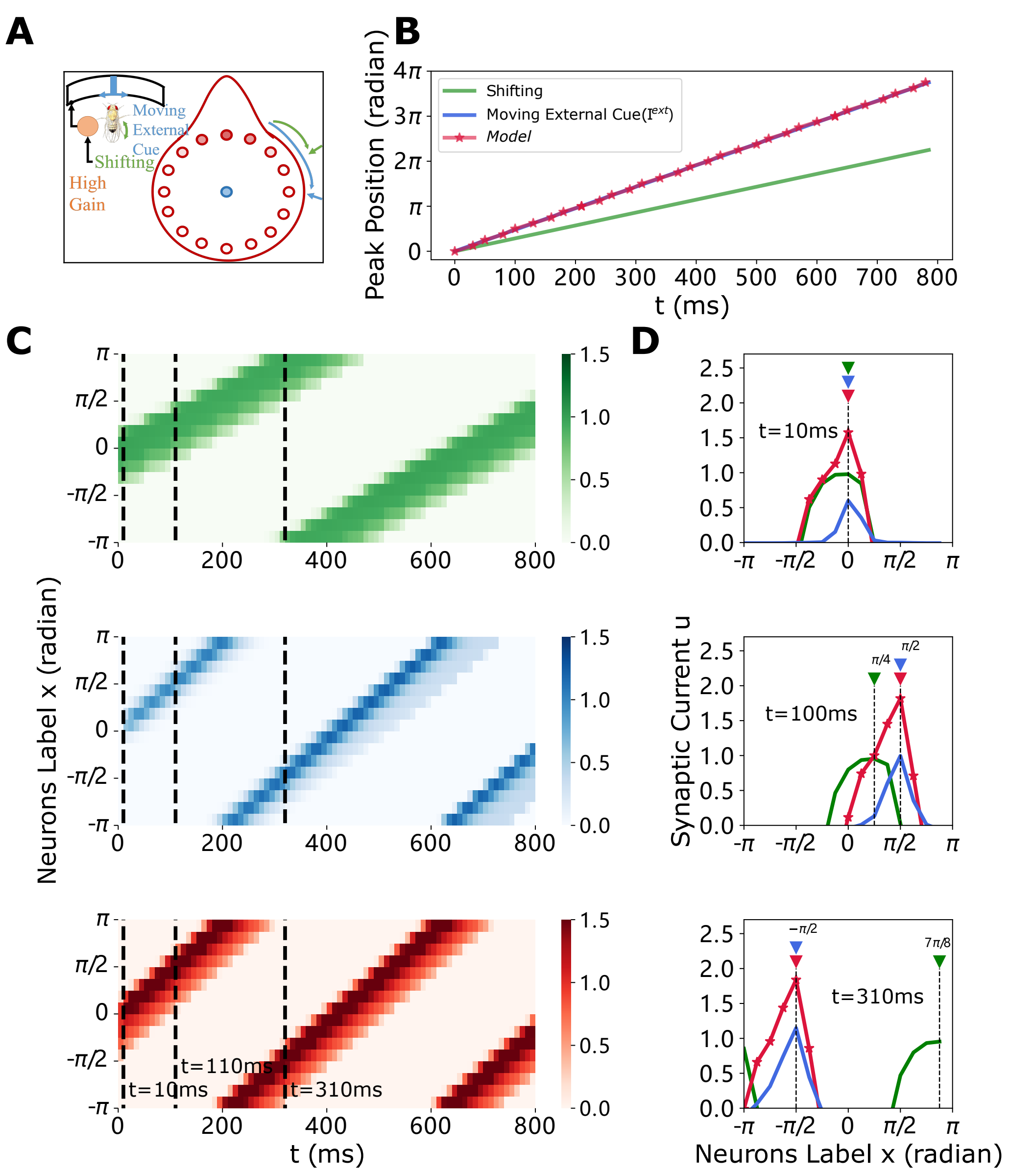

Figure S3: **Traveling activity bumps with external cue input and shifting input in high gain.** A traveling activity bump tracks external cue signal and shifting signal with $c = 0.15$, $\omega = 0.43$, and the initial condition is set to consistent. The color-coding and symbols used here align with the conventions detailed in Fig.S1. (A) The experimental schematic with both inputs in high gain. (B) The peak position propagates at a constant speed with time passing, and ring attractor network tracks moving external cue input almost perfectly. (C) The space-time plot displays the shifting input, external cue input, and the output of the ring attractor network. (D) The profiles of the traveling bump are shown at t=10, 100, 310 ms.

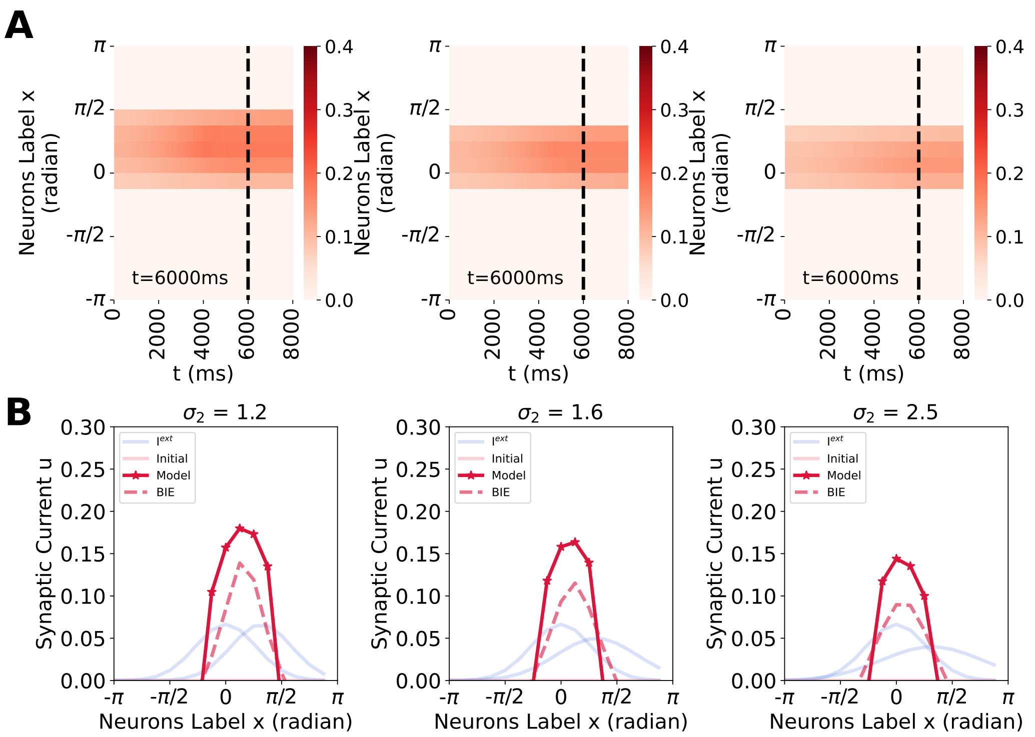

Figure S4: **Bayesian-like integration with varying σ_2.** Parameters are set to $\mu_1 = 0$, $\mu_2 = 1$, $\sigma_1 = 1.2$, and $\sigma_2 = 1.2, 1.4, 1.6$, respectively. The model shows Bayesian-like integration. (A) The space-time plot. (B) The profile plot when t = 6000 ms.

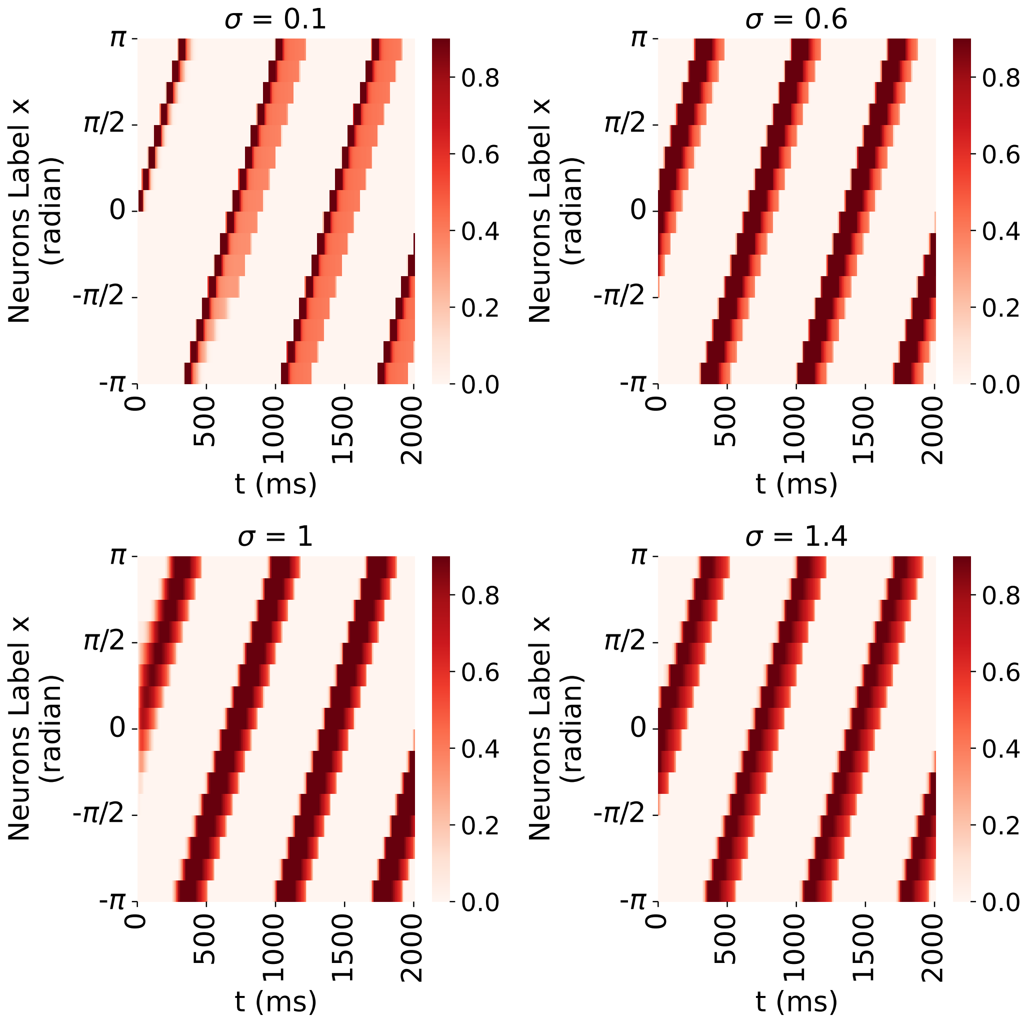

Figure S5: **Traveling bump with varying σ.** The space-time plot. Parameters are set to $\mu = 0$, and $\sigma = 0.1, 0.6, 1, 1.4$, respectively. The model tracks the moving external cue input.

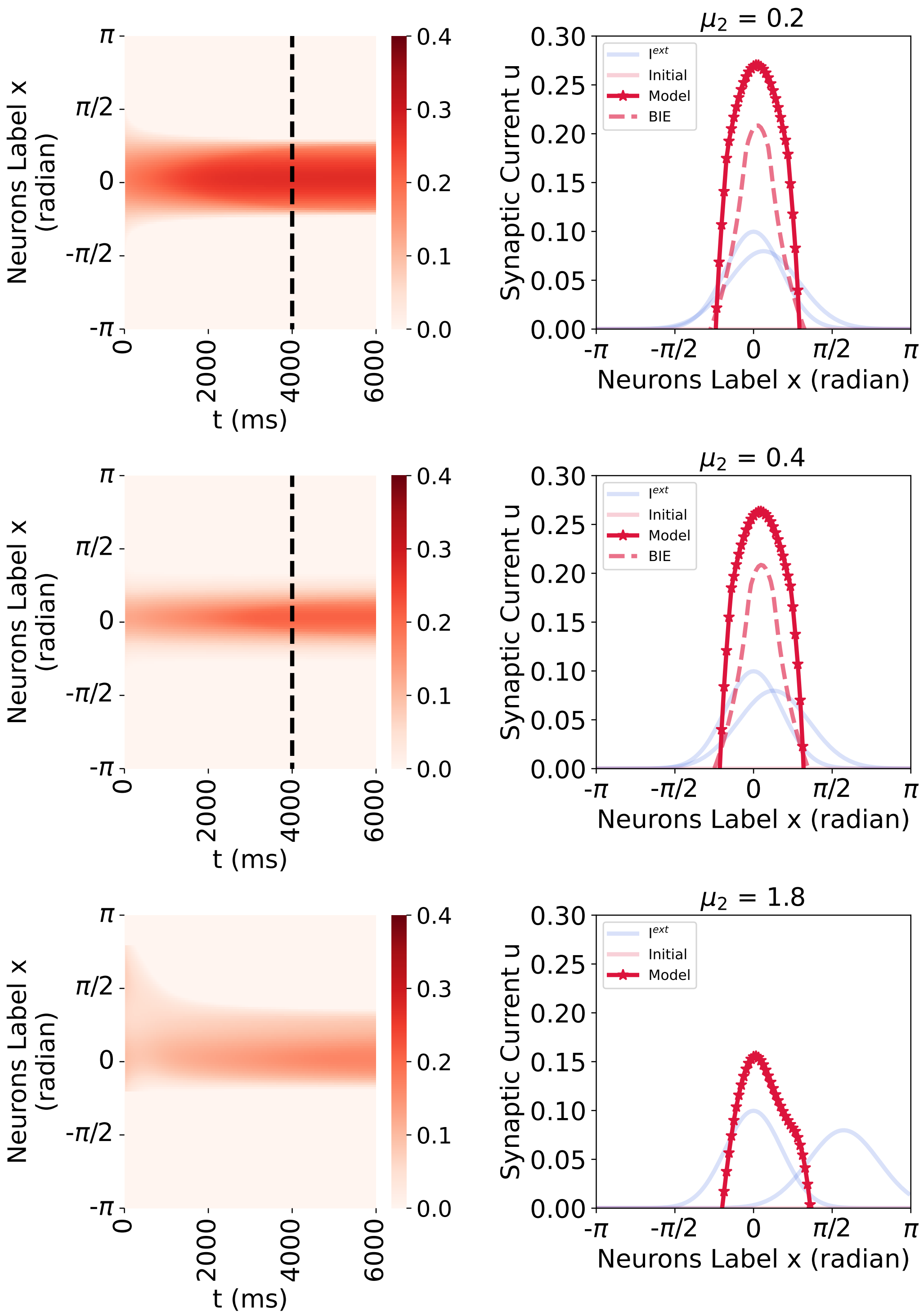

Figure S6: **Bayesian-like integration and WTA-like response with 128 neurons.** Parameters are set to $\mu_1 = 0$, $\sigma_1 = 0.8$, $\sigma_2 = 1$, and $\mu_2 = 0.2, 0.4, 1.8$, respectively. (Left) The space-time plot. (Right) The profiles of the model output and external cue input.

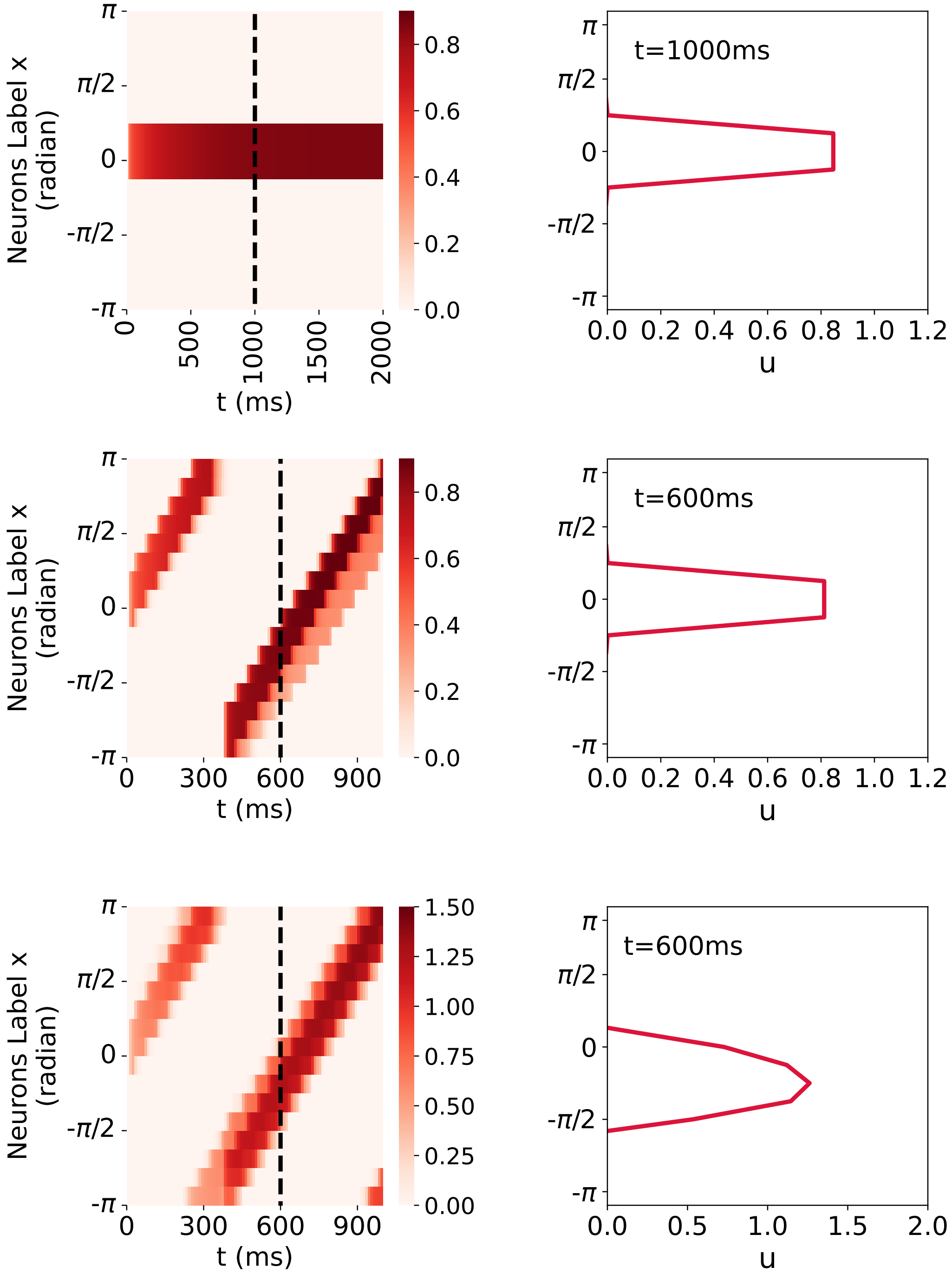

Figure S7: **Performances of the system with square-wave inputs.** The left panel displays space-time plots, while the right panel displays profile plots. (Top) The model demonstrates persistence with square-wave initial condition (no inputs). (Middle) The model tracks external cues, resulting in an output that resembles a square wave. (Bottom) The model continues to track external cues, the output no longer maintains the square wave shape.

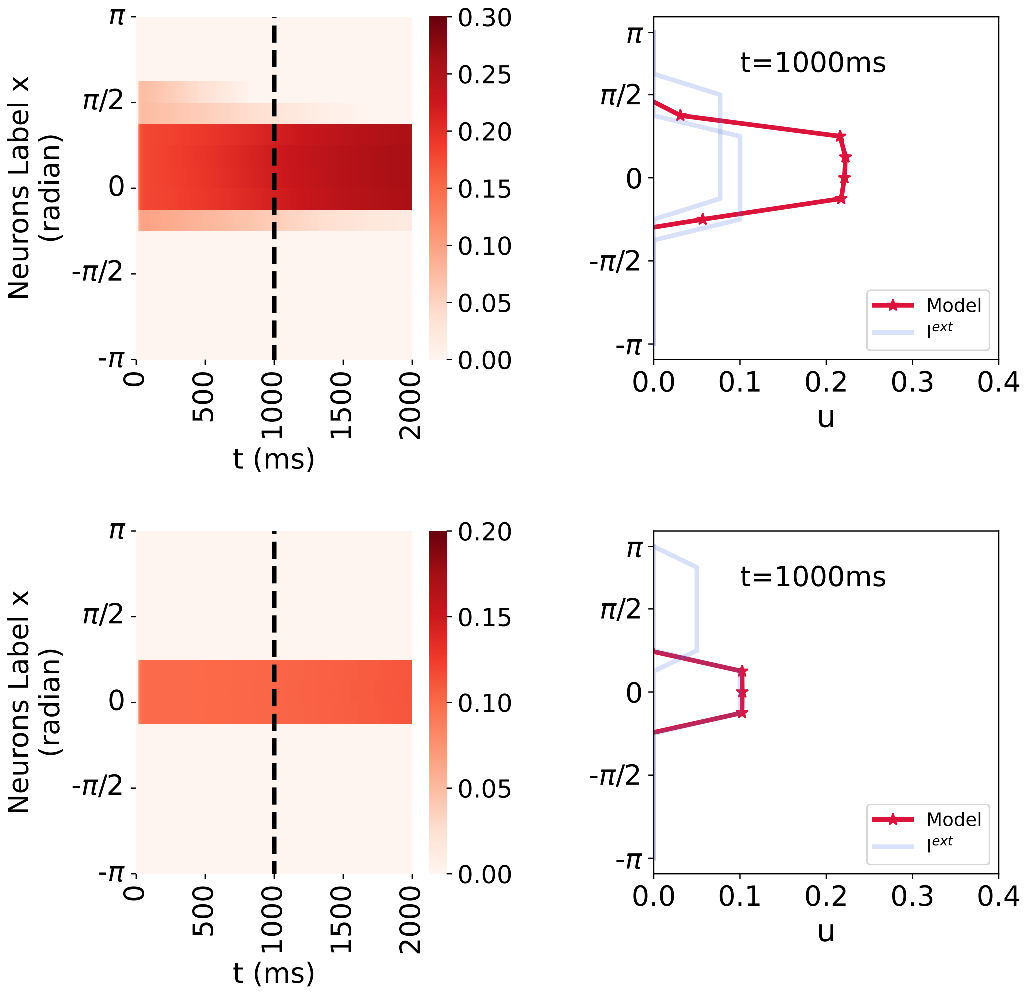

Figure S8: **Integration and WTA response with square-wave inputs.** The left panel displays space-time plots, while the right panel displays profile plots. (Top) The model demonstrates information integration, although the specific type of integration remains unspecified. (Bottom) The model demonstrates WTA response.

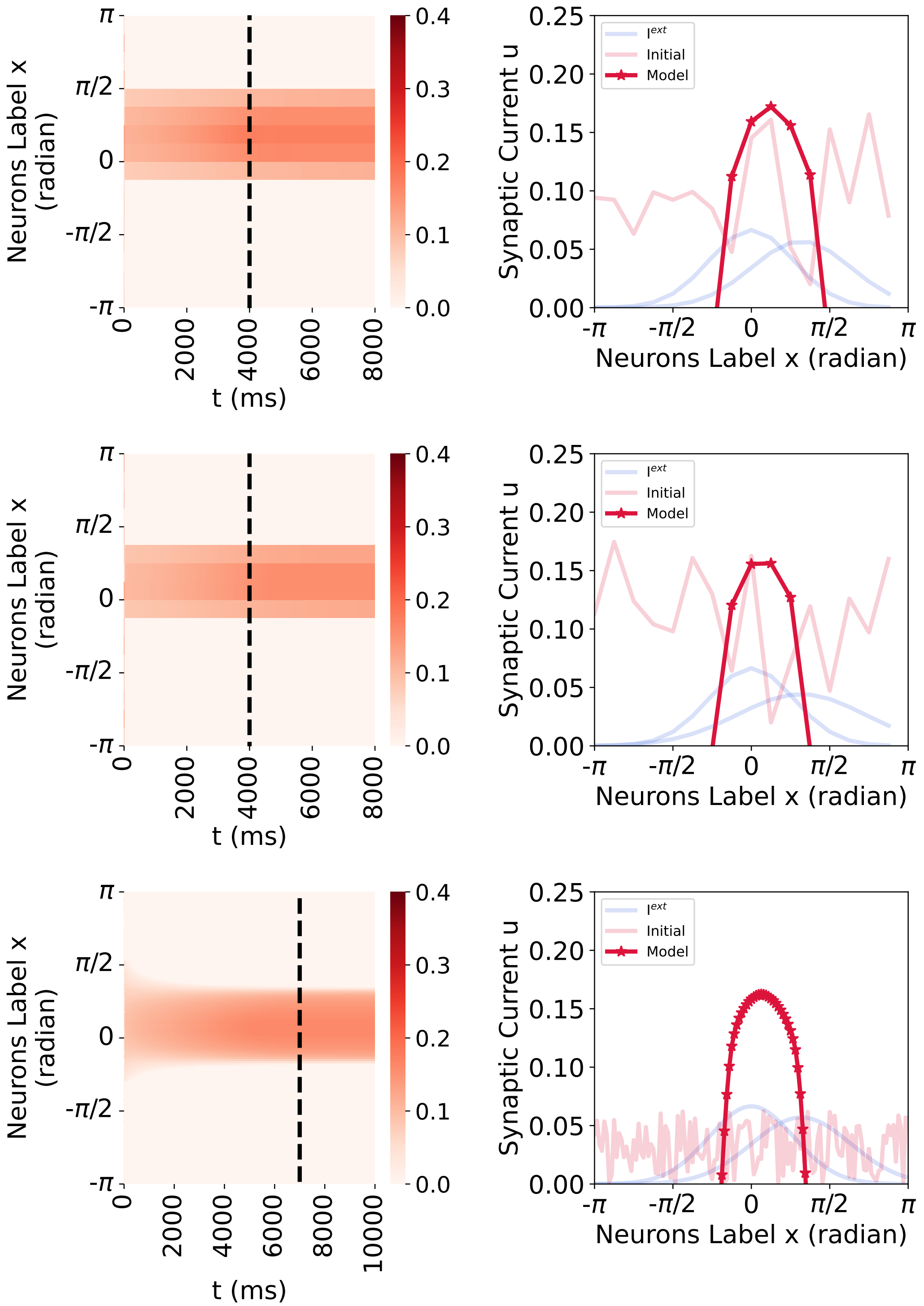

Figure S9: **Stable solution of model with random initial.** The left panel displays space-time plots, while the right panels displays profile plots. (Top, Middle) $N = 16$. (Bottom) $N = 128$. Parameters are set to $\mu_1 = 0$, $\mu_2 = 1$, $\sigma_1 = 0.8$, $\sigma_2 = 1.4, 1.8, 1.4$, respectively.
